# Axial Coordination Induced Electron Delocalization and p‐p Orbital Hybridization in Single‐Atom Catalysts Boosts Zn^2+^ Desolvation for Highly Stable Zn Anode

**DOI:** 10.1002/advs.202518148

**Published:** 2025-11-11

**Authors:** Yan Dao, Mengyuan Li, Miaomiao Zhang, Ke Fan, Qi Qi, Lin Zhang, Xin‐Yao Yu

**Affiliations:** ^1^ School of Materials Science and Engineering Anhui University Hefei 230601 P. R. China; ^2^ Institute of Solid State Physics Leibniz University Hannover 30167 Hannover Germany

**Keywords:** axial coordination, desolvation, electron delocalization, single atoms, zinc anode

## Abstract

Aqueous zinc‐ion batteries have emerged as promising candidates for next‐generation energy storage due to their superior safety and lower cost. However, the sluggish desolvation kinetics of hydrated zinc ions at anode causes critical issues, including dendritic growth, hydrogen evolution reaction (HER), and anode corrosion. Herein, taking Sb single‐atoms (SAs) as a proof‐of‐concept, a F‐axial coordination engineering strategy is proposed to accelerate the desolvation kinetics and thus ensure robust cycling stability of Zn anode. Theoretical calculations reveal that the electron delocalization triggered by F‐axial coordination and p‐p orbital hybridization between Sb and F atoms strengthens the affinity for Zn. Electrochemical analysis and in situ/ex situ spectroscopy characterizations demonstrate that the introduction of F‐axial coordination can effectively reduce the energy barrier for the desolvation process of hydrated zinc ions, inhibit HER, uniform Zn^2+^ diffusion, and enable lateral Zn deposition. Consequently, the symmetric zinc battery based on F‐axial coordinated Sb SAs (F‐Sb SAs)‐modified Zn anode features unprecedented stability up to 6000 and 1200 h at 5 and 20 mA cm^−2^, respectively. Furthermore, the fabricated F‐Sb SAs@Zn||I_2_ battery exhibits remarkably long durability at a high current density of 10 A g^−1^, maintaining 94.3% of capacity even after 100 000 cycles.

## Graphical abstract

The F‐axial coordinated Sb SAs (F‐Sb SAs) can effectively reduce the desolvation energy of hydrated Zn^2+^ ions and greatly accelerate the migration kinetics of Zn^2+^ ions. The symmetric cell and Zn‐I_2_ batteries based on F‐Sb SAs@Zn anode exhibit outstanding stability.

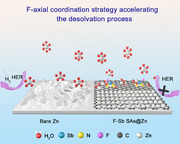

## 1 Introduction

Aqueous zinc‐ion batteries (AZIBs) show great potential for applications in large‐scale grid energy storage, owing to their high theoretical specific capacity (820 mAh g^−1^),^[^ ^1^, ^2^ ^]^ low redox potential (−0.763 V vs. standard hydrogen electrode), and environmental compatibility.^[^ ^3^, ^4^, ^5^, ^6^, ^7^, ^8^ ^]^ However, the practical application of Zn anodes is largely hindered by unfavorable Coulombic efficiency (CE) and poor cycling stability, primarily stemming from some major challenges including hydrogen evolution reaction (HER),^[^ ^9^ ^]^ anode corrosion,^[^ ^10^ ^]^ by‐product formation,^[^ ^11^ ^]^ and dendrite growth.^[^ ^12^, ^13^, ^14^, ^15^ ^]^ These bottlenecks are largely caused by the sluggish desolvation kinetics of hydrated zinc ions.^[^ ^16^, ^17^ ^]^

In aqueous electrolyte, Zn^2+^ ions predominantly form [Zn(H_2_O)_6_]^2+^ complexes.^[^ ^18^, ^19^ ^]^ Prior to deposition, Zn^2+^ ions must be completely detached from its tightly bound solvation shell.^[^ ^20^ ^]^ Due to the strong coordination between Zn^2+^ and water molecules, only partially dehydrated [Zn(H_2_O)*x*]^2+^ (*x* < 6) or those accompanied by residual water molecules participate in the deposition reaction, leading to uneven distribution of Zn^2+^ and inhomogeneous Zn deposition and thus favoring the growth of dendrites.^[^ ^21^ ^]^ Moreover, the incomplete desolvated [Zn(H_2_O)*x*]^2+^ or adsorbed water molecules on the electrode surface, act as HER mediators and accept electrons from the metallic zinc surface, resulting in severe HER.^[^ ^22^, ^23^ ^]^ The HER consumes the charges which would otherwise be used for zinc deposition, thereby reducing the CE.^[^ ^24^ ^]^ On the other hand, it also increases the local pH, which promotes the formation of insulating byproducts such as Zn_4_SO_4_(OH)_6_·5H_2_O on the anode surface.^[^ ^25^, ^26^, ^27^ ^]^ These issues collectively shorten the cycle life of Zn anode. In fact, the desolvation of hydrated zinc ions can be conceptualized as a dissociation reaction featuring a distinct energy barrier.^[^ ^4^, ^17^, ^21^, ^28^, ^29^ ^]^ Consequently, the desolvation kinetics of hydrated zinc ions can be tailored by incorporating active catalysts into the systems.

With the merits of 100% atomic utilization and high catalytic activity, single‐atoms (SAs)‐based catalysts have garnered widespread attention in the field of energy storage and conversion.^[^ ^30^, ^31^, ^32^ ^]^ In lithium‐metal batteries, SAs have been utilized as electrocatalysts to promote the dissociation of the Li^+^ solvation structure to release free Li ions.^[^ ^33^, ^34^, ^35^ ^]^ However, the exploration of SAs‐based catalysts to accelerate the desolvation kinetics of hydrated zinc ions in AZIBs is rarely reported. Although previous works demonstrate that SAs‐based materials perform well as protection layer for dendrite‐free zinc anode, SAs are regarded as atomic deposition sites for Zn.^[^ ^36^, ^37^ ^]^

Modulating the coordination of SAs is an effective strategy to regulating their catalytic performance.^[^ ^38^, ^39^ ^]^ As a new regulation approach, axial coordination engineering is demonstrated to be highly efficient in tailoring the electronic structure of metal centers, achieving control over the intrinsic activity.^[^ ^40^, ^41^, ^42^ ^]^ For instance, Wang's group found that the introduction of axial Cl ligand to single‐atom Fe‐N_4_ catalyst can optimize the adsorption free energy of OH intermediates, significantly enhancing the oxygen reduction reaction process.^[^ ^43^ ^]^ Previous studies have shown that axial ligand coordination at single‐atom centers is capable of inducing electron redistribution and orbital reconfiguration to enhance the activity of SAs.^[^ ^44^, ^45^ ^]^ Therefore, we predict that axially coordinated SAs hold great potential in promoting the desolvation kinetics of hydrated zinc ions.

Herein, antimony‐N_4_ SAs (denoted as Sb SAs) is employed as a conceptual promoter to improve the desolvation kinetics of hydrated Zn^2+^ ions. With narrower valence bandwidths, Sb is more susceptible to form high‐valence states during catalytic reactions, thus elevating the reaction rate.^[^ ^46^, ^47^ ^]^ We introduce a fluorine (F)‐axial coordination strategy to accelerate the desolvation process. The effects of F‐axial coordination on the rapid desolvation of hydrated zinc ions for attaining a highly stable zinc anode are characterized by comprehensive electrochemical analysis, in situ optical microscopy, in situ Raman spectroscopy, as well as density functional theory (DFT) calculations and COMSOL simulations. As expected, the F‐Sb SAs‐modified Zn anode features the capability to facilitate the migration process of Zn^2+^, promote the nucleation kinetics of Zn, and inhibit the HER and other side reactions. Moreover, in both Zn symmetric batteries and full cells with I_2_‐loaded activated carbon (AC) as cathode, the F‐Sb SAs@Zn anode manifests impressive stability.

## 2 Results and Discussion

**Figure 1:**
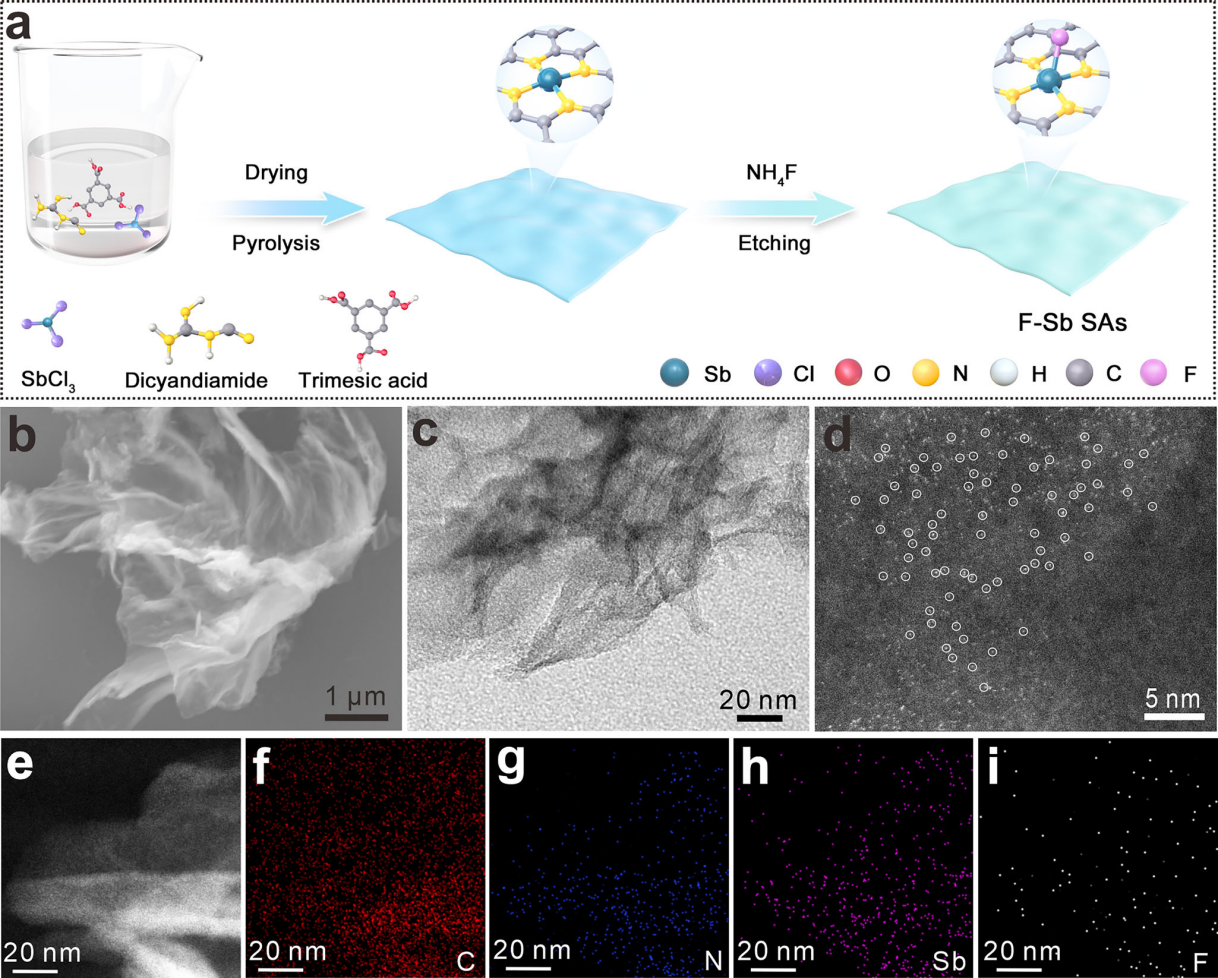
a) Schematic illustration for the preparation procedure of F‐Sb SAs. b) SEM image, c) TEM image, d) magnified HAADF‐STEM image, and e–i) EDS elemental mapping images of F‐Sb SAs.

The F‐Sb SAs is synthesized through a two‐step pyrolysis approach, as depicted in **Figure** **1**a. The homogenized precursors are first pyrolyzed, followed by NH_4_F‐mediated fluorination and acid etching processes. This approach yields material with amorphous carbon structure and graphene‐like morphology (Figure 1b; Figure S1a,b, Supporting Information). As revealed by energy‐dispersive spectroscopy (EDS), both Sb and F elements are incorporated into the carbon material. The introduced fluorine is ≈ 1.51 at% (Figure S1c, Supporting Information). No particles are detected in the transmission electron microscopy (TEM) image (Figure 1c), while high‐angle annular dark‐field scanning TEM (HAADF‐STEM) image reveals abundant bright spots (Figure 1d), probably originating from isolated Sb atoms. The EDS elemental mapping images also demonstrate the existence of Sb, C, N, and F elements in the product (Figure 1e–i). The loading of Sb is determined to be as high as 11.6 wt.% by inductively coupled plasma optical emission spectrometry. As control samples, Sb SAs without the introduction of fluorine, N‐doped carbon (NC), and F,N co‐doped carbon (FNC) are also synthesized using similar synthetic methods (Figures S2–S4, Supporting Information).

**Figure 2:**
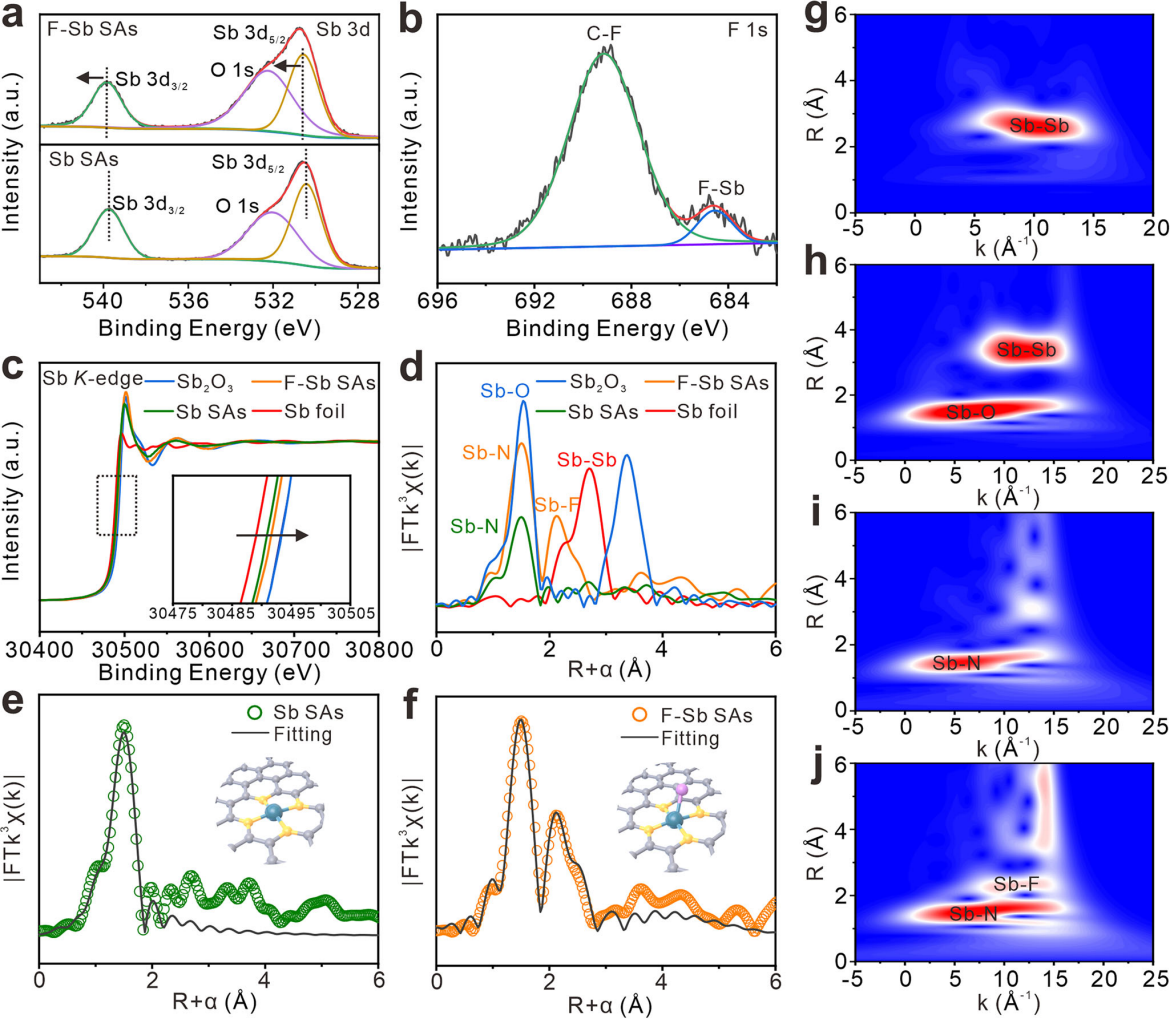
a) Sb 3d XPS spectra of F‐Sb SAs and Sb SAs. b) F 1s XPS spectra of F‐Sb SAs. c) Sb *K*‐edge XANES spectra and d) FT‐EXAFS spectra of Sb_2_O_3_, Sb foil, Sb SAs, and F‐Sb SAs. e,f) The EXAFS fitting data of the F‐Sb SAs and Sb SAs based on the models obtained from DFT optimization. The insets show optimized molecular models based on DFT. g–j) WT‐EXAFS plots of Sb foil, Sb_2_O_3_, Sb SAs, and F‐Sb SAs.

X‐ray photoelectron spectroscopy (XPS) and extended X‐ray absorption fine structure (EXAFS) measurements are performed to investigate the composition, chemical state, and coordination environment of the two Sb‐containing samples. The emergence of F signal in the XPS survey spectrum, further verifying the introduction of fluorine in the final product (Figure S5a, Supporting Information). The detected C─F bonding in the C 1s spectrum confirms the insertion of F into the carbon matrix (Figure S5b,c, Supporting Information). The N 1s spectra also identify the doping of N element in carbon (Figure S5d,e, Supporting Information). As shown in **Figure** **2**a, the incorporation of F triggers the shift of Sb 3d spectrum to higher energy, suggesting that the F element increases the valence state Sb.^[^ ^48^ ^]^ Apart from C─F bond, the peak at ≈ 684.56 eV corresponding to F‐Sb appears (Figure 2b),^[^ ^49^ ^]^ verifying the coordination between F and Sb. The Sb *K*‐edge X‐ray absorption near‐edge structure (XANES) spectra (Figure 2c) show that both materials exhibit absorption energies between Sb foil and Sb_2_O_3_, confirming Sb^δ+^ oxidation states (0< δ <3). The F‐containing material displays a 1.7 eV of positive shift in the white‐line (inset of Figure 2c), evidencing elevated Sb valence state, consistent with XPS result. The Fourier‐transformed (FT) EXAFS spectra in Figure 2d further identifies dominant coordination peaks at ≈ 1.58 Å (Sb‐N) and 2.06 Å (Sb‐F) with no detectable Sb‐Sb scattering, proving the formation of SAs. In contrast, the material without fluorination presents only one major peak of Sb─N bond. By combining EXAFS fitting and structural modeling results (Figure 2e,f; Figures S6 and S7 and Table S1, Supporting Information), the configuration of the two samples is determined to be Sb‐N_4_ and Sb‐N_4_ with F‐axial coordination. As such, the final product is named as F‐Sb SAs, while the material without F is named as Sb SAs. The wavelet‐transformed (WT)‐EXAFS further unambiguously confirm the successful synthesis of atomically dispersed SbN_4_‐F motif with axial fluorine coordination (Figure 2g–j).

**Figure 3:**
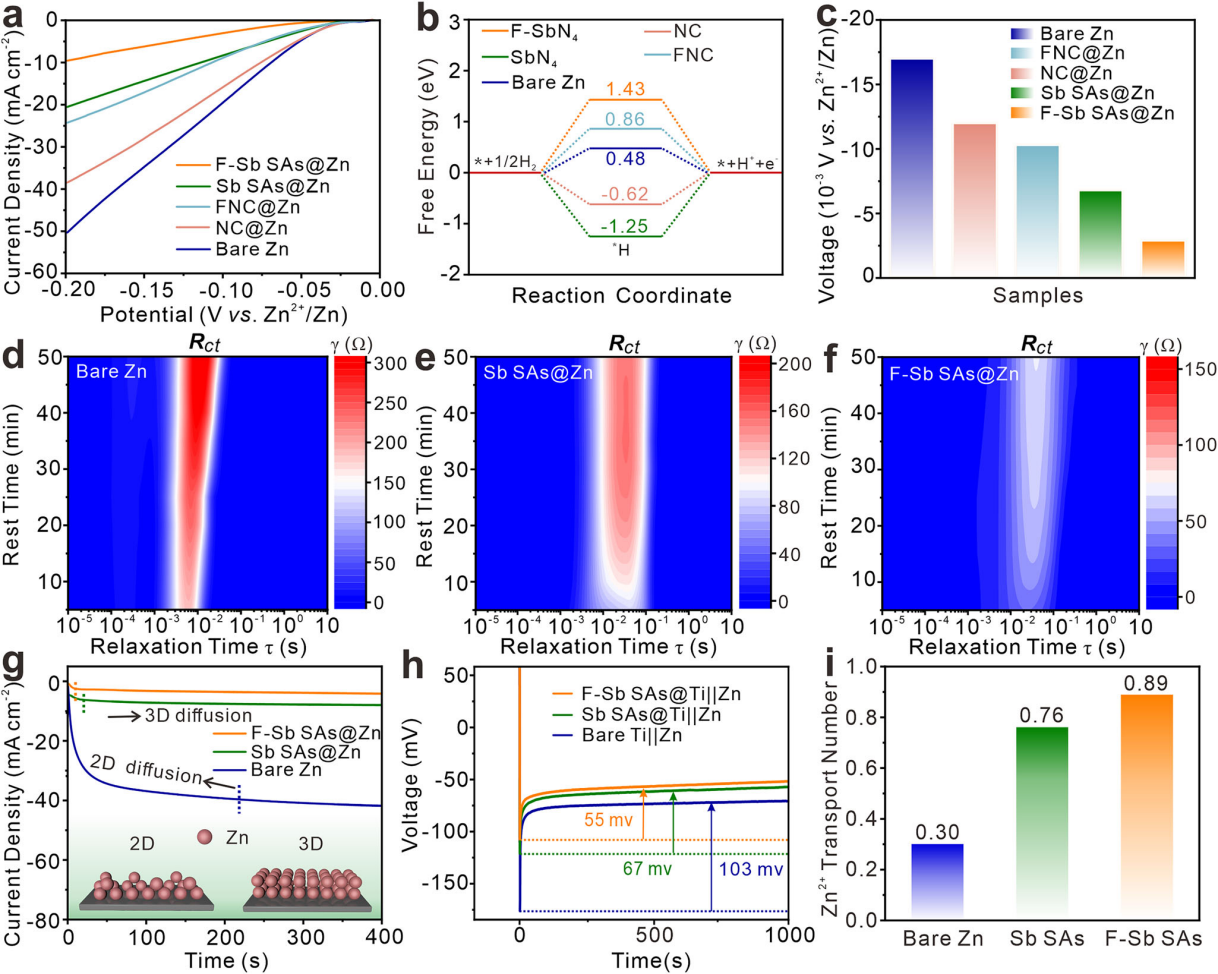
a) LSV curves, b) Gibbs free energy diagrams of hydrogen adsorption, and c) corrosion potentials of bare Zn, NC@Zn, F‐NC@Zn, Sb SAs@Zn, and F‐Sb SAs@Zn tested in two‐electrode system. (d‐f) DRT analysis of bare Zn, Sb SAs@Zn and F‐Sb SAs@Zn in symmetric cells during standing by EIS. (g) CA curves at an overpotential of −150 mV. h) Voltage‐time curves of Zn plating on bare Ti||Zn, F‐Sb SAs@Ti||Zn, and Sb SAs@Ti||Zn. i) Comparison for Zn^2+^ transport number of bare Zn, Sb SAs@Zn, and F‐Sb SAs@Zn.

All the synthesized materials are then coated onto Zn foil to evaluate their performance enhancement on Zn anodes (Figure S8, Supporting Information). The linear sweep voltammetry (LSV) curves (**Figure** **3**a; Figure S9a, Supporting Information) show that the HER inhibition trend of these materials is F‐Sb SAs > Sb SAs > FNC > NC. To explore the origin for this trend, DFT calculations are carried out (Figure S10, Supporting Information). As shown in Figure 3b, the F‐axial coordinated SbN_4_ (F‐SbN_4_) model exhibits the largest hydrogen adsorption Gibbs free energy (|Δ*G*_H_| = 1.43 eV) among the five systems, verifying its superior ability to suppress HER. Notably, F‐axial coordination can enhance HER inhibition effectively, as evidenced by the higher |Δ*G*_H_| values of F‐SbN_4_ model than that of SbN_4_ model. In addition, the large |Δ*G*_H_| value of F,N co‐doped carbon model over that of N‐doped carbon model also indicates the constructive impact of F‐doping in mitigate HER. Hence, the synergistic function of F‐doping and F‐axial coordination enables the F‐Sb SAs with the best inhibition capability for HER. The inhibition of HER is further demonstrated by differential electrochemical mass spectrometry (DEMS) during the Zn plating/stripping (Figure S11, Supporting Information). Obviously, substantial H_2_ evolution is observed on bare Zn but not on F‐Sb SAs@Zn.

Meanwhile, the F‐Sb SAs@Zn also illustrates higher corrosion potential than Sb SAs@Zn, FNC@Zn, NC@Zn, and bare Zn anodes, as determined by the Tafel plots (Figure 3c; Figures S9b and S12, Supporting Information). When immersing bare Zn, Sb SAs@Zn, and F‐Sb SAs@Zn in 2 M ZnSO_4_ electrolyte for 7 and 14 days, the corrosion products of Zn_4_SO_4_(OH)_6_·5H_2_O with inhomogeneous flaky structure appear on both bare Zn and Sb SAs@Zn surfaces, while negligible Zn_4_SO_4_(OH)_6_·5H_2_O can be found on the surface of F‐Sb SAs@Zn (Figure S13, Supporting Information). Additionally, the F‐Sb SAs coating remains well‐adhered to the zinc substrate after immersion (Figure S14, Supporting Information). The highly negative Zeta potential in water and more positive Zeta potential in 2 m ZnSO_4_ for F‐Sb SAs indicate that F‐Sb SAs show great capability to repel the corrosion of anions such as OH^−^ and SO_4_^2−^ (Figure S15, Supporting Information).^[^ ^50^ ^]^ Furthermore, the distribution of relaxation time (DRT) analysis of operando EIS during resting is carried out (Figure 3d–f; Figure S16, Supporting Information). In the DRT plots, the intensities of *R*_ct_ for both bare Zn and Sb SAs@Zn increase rapidly with resting time, suggesting the continuous accumulation of byproducts at the electrode‐electrolyte interface. In sharp contrast, F‐Sb SAs@Zn maintains a low impedance, demonstrating its excellent interface stability. The enhanced corrosion resistance may be partially attributed to the superior hydrophobic surface property of F‐Sb SAs@Zn (Figure S17, Supporting Information).

Chronoamperometry (CA) is then utilized to evaluate the nucleation dynamics (Figure 3g; Figure S18, Supporting Information). Bare Zn requires a long 2D diffusion time of 220 s for Zn^2+^ ions to across the anode surface (Figure 3g). Surprisingly, only 9 s is needed for F‐Sb SAs@Zn anode to complete 2D to 3D diffusion transition, much shorter than that of Sb SAs@Zn anode (20 s), FNC@Zn anode (26 s), and NC@Zn anode (70 s), manifesting an accelerated Zn^2+^ deposition kinetics on the surface of F‐Sb SAs@Zn electrode. The better performance of F‐Sb SAs@Zn and Sb SAs@Zn anodes than FNC@Zn and NC@Zn anodes validates the effective role of SAs. Additionally, the enhanced efficacy of fluorinated Sb SAs over unmodified Sb SAs counterparts and similarly of FNC versus NC both highlight the beneficial effect of F‐incorporation. The voltage gap, corresponding to the driving force between nucleation and growth processes, is effectively reduced from 103 mV for bare Zn to 55 mV for F‐Sb SAs, 67 mV for Sb SAs, 72 mV for FNC, and 75 mV for NC (Figure 3h; Figure S19, Supporting Information).^[^ ^51^ ^]^ The advantage of F‐Sb SAs in lowering nucleation overpotential is evidenced in cyclic voltammetry (CV) tests (Figure S20, Supporting Information). Based on CA and electrochemical impedance spectroscopy (EIS) results (Figure 3i; Figure S21, Supporting Information), the calculated Zn^2+^ transfer number (*t*_Zn2+_) in the electrode/electrolyte interface of F‐Sb SAs@Zn (0.89) outperforms that of Sb SAs@Zn (0.76), FNC@Zn (0.61), NC@Zn (0.53), and bare Zn (0.30), which can be attributed to the accelerated desolvation kinetics.

**Figure 4:**
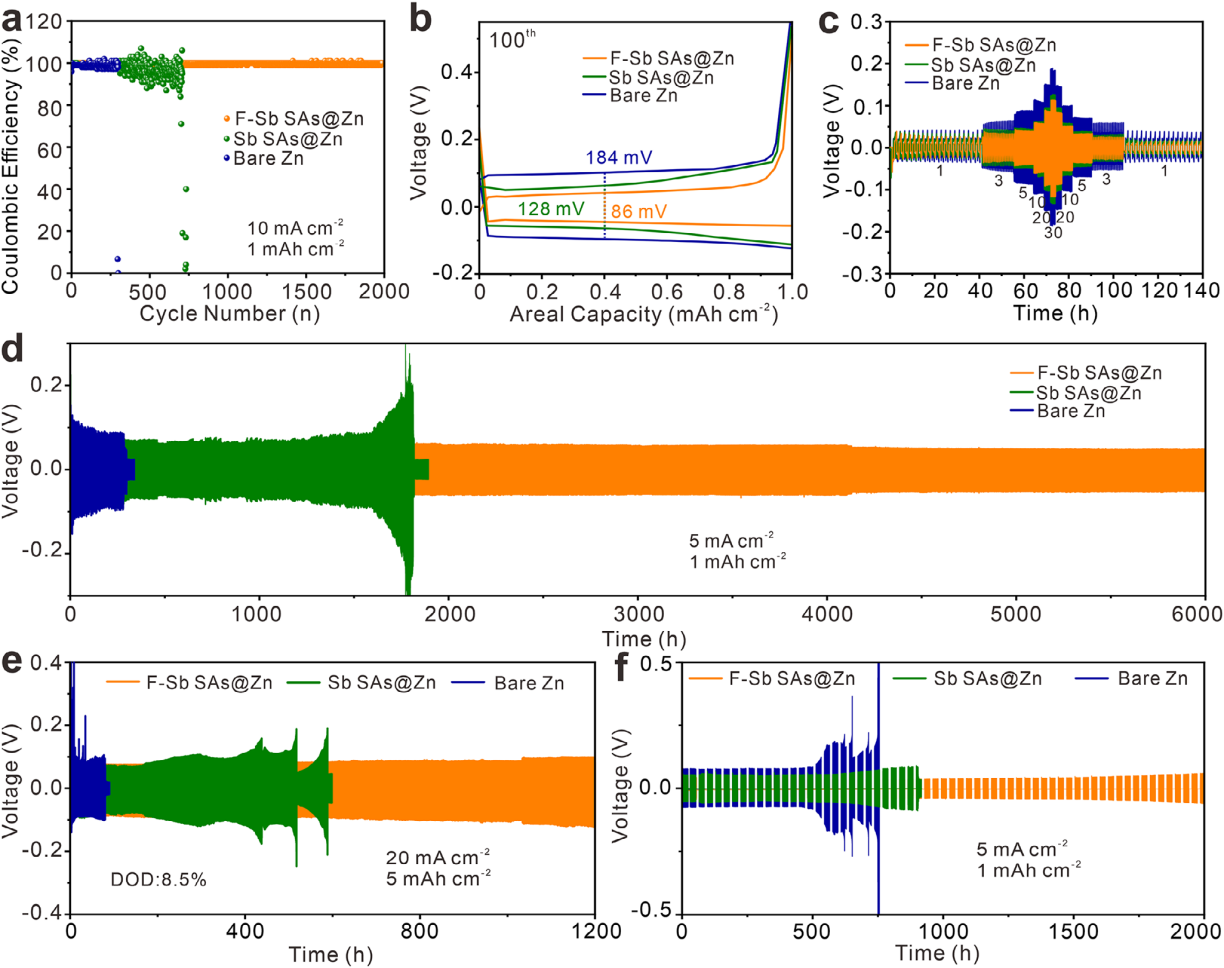
a) CE profiles of Zn||Ti, Sb SAs@Zn||Ti, and F‐Sb SAs@Zn||Ti at 10 mA cm^−2^ and 1 mAh cm^−2^. b) Galvanostatic charge/discharge curves of Zn||Ti, Sb SAs@Zn||Ti, and F‐Sb SAs Zn||Ti. c) Rate performance at different current densities from 1 to 30 mA cm^−2^ of bare Zn, Sb SAs@Zn, and F‐Sb SAs@Zn. (d) Long‐term cycling stability of bare Zn, Sb SAs@Zn, and F‐Sb SAs@Zn symmetric cells at 5 mA cm^−2^ and 1 mAh cm^−2^. e) Cycling stability at 20 mA cm^−2^ and 5 mAh cm^−2^. f) The shelving‐recovery performance of bare Zn, Sb SAs@Zn, and F‐Sb SAs@Zn under 5 mA cm^−2^ and 1 mA h cm^−2^.

Next, the Zn plating and stripping reversibility of various anodes are systematically evaluated. The CE of the Zn||Ti and Sb SAs@Zn||Ti cells decline notably after 252 and 724 cycles, respectively, under a current density of 10 mA cm^−2^ and an areal capacity of 1 mAh cm^−2^. In stark contrast, the F‐Sb SAs@Zn||Ti cell maintains an exceptionally high average CE of 99.36% over 2000 cycles (**Figure** **4**a). In addition, a high and stable CE is also exhibited by the F‐Sb SAs@Zn||Ti cell at various current densities (Figure S22, Supporting Information). Correspondingly, as observed from the voltage‐capacity profiles after 100 cycles (Figure 4b), the average voltage gap of F‐Sb SAs@Zn||Ti cell (86 mV) is narrower than that of the Zn||Ti cell (184 mV) and the Sb SAs@Zn||Ti cell (128 mV), reflecting the enhanced Zn plating/stripping kinetics on F‐Sb SAs@Zn||Ti electrode. The rate performance tests reveal that the F‐Sb SAs@Zn symmetric cell can deliver a smaller voltage hysteresis and better stability than the cells based on Sb SAs@Zn and bare Zn under different current densities ranging from 1 to 30 mA cm^−2^ (Figure 4c). The superior rate performance of F‐Sb SAs@Zn can be attributed to the small polarization on it. As elucidated by galvanostatic intermittent titration technique (GITT) tests, compared to Sb SAs and bare Zn, the F‐Sb SAs can more effectively mitigate concentration polarization, ohmic and interfacial electrochemical polarization (Figure S23, Supporting Information).

As anticipated, the F‐Sb SAs@Zn electrode achieves a substantially prolonged lifespan of 6000 h at 5 mA cm^−2^ and 1 mAh cm^−2^ with a cumulative capacity as high as 15 000 mAh cm^−2^ (Figure 4d), far exceeding bare Zn (296 h) and Sb SAs@Zn (1800 h). Even tested under more severe condition (20 mA cm^−2^ and 5 mAh cm^−2^), the F‐Sb SAs@Zn cell still presents superior cycling stability up to 1200 h with 8.5% of DOD (Figure 4e), again demonstrating the high durability and reversibility of F‐Sb SAs@Zn. Even under a higher DOD of 17%, robust performance is also demonstrated by the F‐Sb SAs@Zn anode (Figure S24, Supporting Information). As confirmed by XPS and SEM, the F─Sb bond can be still observed in F‐Sb SAs after cycling and the electrode maintains intact, suggesting good structural stability (Figure S25, Supporting Information). Notably, without single‐atom Sb, the FNC@Zn and NC@Zn electrodes show inferior cycling durability (Figure S26, Supporting Information). The concentration of incorporated fluorine and the coating thickness also greatly influence the cycling performance and are optimized to 1.51 at% and 12.53 µm, respectively (Figures S27 and S28, Supporting Information). Remarkably, the optimal F‐Sb SAs@Zn anode also surpasses many previously reported Zn anodes in terms of cumulative capacity and cycle life (Table S2, Supporting Information). Furthermore, the shelving‐recovery tests are conducted to evaluate the practical applicability (Figure 4f). Withstanding repeated steps in working and shelving each lasting 12 h, bare Zn persists for less than 600 h before experiencing a short circuit, signifying the severe corrosion reactions during long periods of quiescent state. On the contrary, the F‐Sb SAs@Zn cell operates over 2000 h with stable polarization voltages, suggesting the splendid property of F‐Sb SAs on inhibiting side reactions.

**Figure 5:**
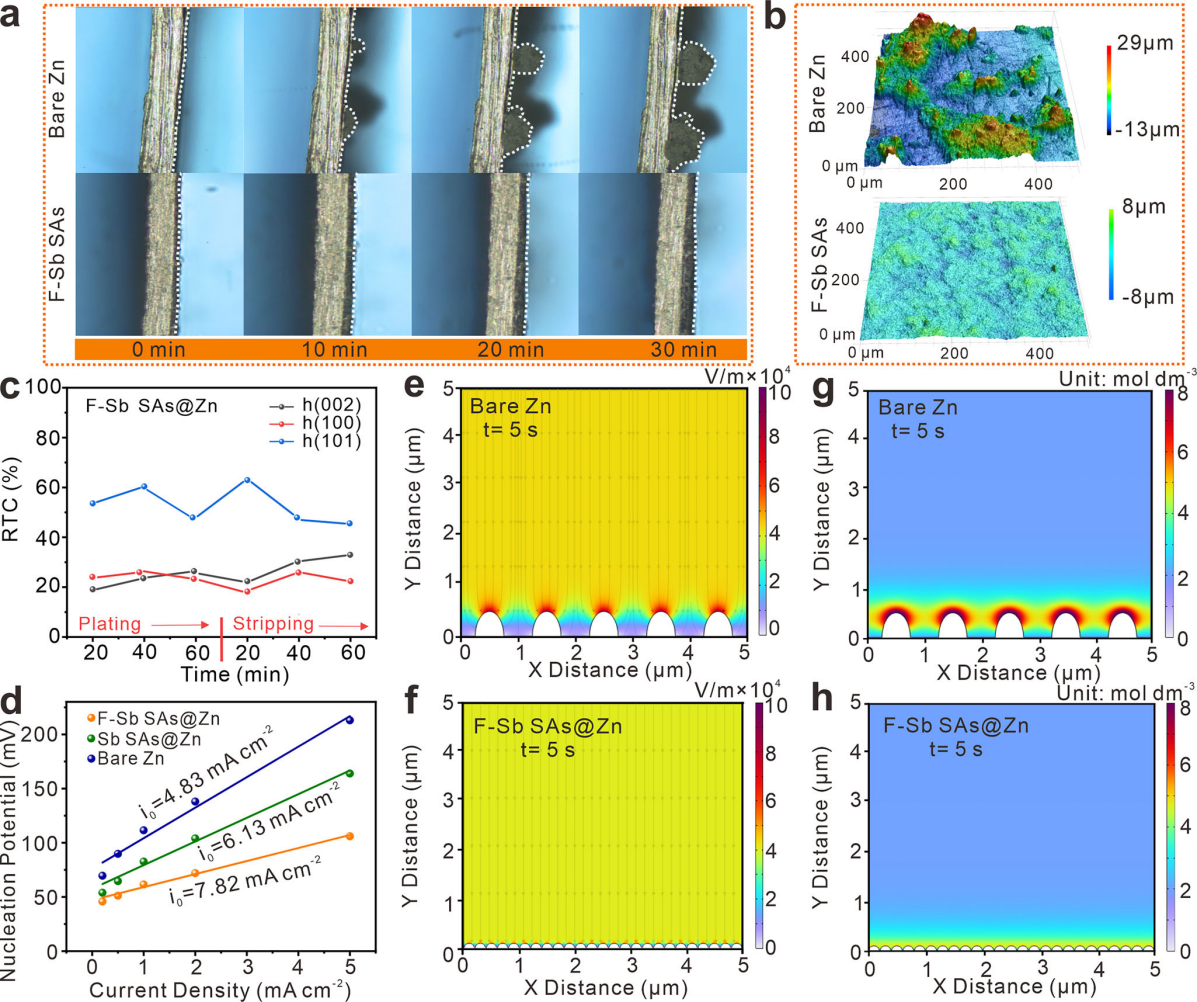
a) In situ optical microscope images of bare Zn and F‐Sb SAs@Zn during plating at 5 mA cm^−2^. b) 3D CLSM images of bare Zn and F‐Sb SAs@Zn after 100 cycles at 5 mA cm^−2^ and 1 mAh cm^−2^. c) Line charts of fitted RTCs for (002), (100), and (101) Zn planes of F‐Sb SAs@Zn. (d) Exchange current density of bare Zn, Sb SAs@Zn, and F‐Sb SAs@Zn electrodes. COMSOL finite element simulation of electric field distribution (e,f) and Zn^2+^ concentration g,h) for bare Zn and F‐Sb SAs@Zn anodes in the electroplating process.

In situ optical microscopy is employed to directly observe the morphology of Zn deposition at the electrolyte/anode interface. As shown in **Figure** **5**a distinct sharp protuberances are observed on bare Zn anode, indicating the formation of Zn dendrite. By contrast, dendrite growth is substantially restricted by the F‐Sb SAs modified Zn anode. This phenomenon is further corroborated by SEM characterization of the electrodes after Zn deposition at 5 mA cm^−2^ (Figure S29, Supporting Information). The Zn mainly deposits on the surface of F‐Sb SAs (Figure S30, Supporting Information). Additionally, in situ DRT analysis during Zn deposition reveals that the F‐Sb SAs@Zn anode presents a shorter relaxation time (Figure S31, Supporting Information), again validating the rapid desolvation kinetics and fast Zn deposition. Furthermore, confocal laser scanning microscopy (CLSM) is employed to provide a macroscopic 3D reconstruction of the cycled Zn anodes (Figure 5b). After 100 cycles, the cycled bare Zn shows pronounced Zn protrusions, resulting in a surface roughness of ≈ 20 µm (Figure S32, Supporting Information). In comparison, the cycled F‐Sb SAs@Zn exhibits relatively smooth Zn deposition, featuring an obviously reduced surface roughness of only 4.5 µm. In addition, distinct characteristic peaks of Zn_4_(OH)_6_SO_4_·5H_2_O by‐product are detected on bare Zn (Figure S33, Supporting Information), while negligible diffraction peaks of undesired product are evident on the F‐Sb SAs@Zn electrode, confirming the efficacy of F‐Sb SAs in suppressing side reactions.

Ex situ XRD patterns are also recorded to investigate the Zn plating/stripping process (Figure S34a,b, Supporting Information). After one hour of plating and stripping, the disordered variation of relative texture coefficient (RTC) value for bare zinc suggests poor reversibility and irregular crystal changes (Figure S34c, Supporting Information). Regarding F‐Sb SAs@Zn, the increase RTC value of preferential (002) plane is detected (Figure 5c).^[^ ^52^ ^]^ In addition, the exchange current density of F‐Sb SAs@Zn is higher than that of the other two electrodes (Figure 5d; Figure S35, Supporting Information), reflecting that F‐Sb SAs can promote surface charge transfer kinetics. This can be due to the high ionic conductivity of the F‐Sb SAs interfacial layer (Figures S36 and S37, supporting information). Finite element simulations are conducted using COMSOL Multiphysics to evaluate the impact of F‐Sb SAs layer on the electric field and Zn^2+^ distribution at the Zn anode interface. As displayed in Figure 5e, a highly inhomogeneous electric field is observed on the surface of bare Zn, leading to a distinct concentration gradient (Figure 5f; Figure S38a, Supporting Information). Following the F‐Sb SAs modification, the electric field and ion concentration become uniformly distributed (Figure 5g,h; Figure S38b, Supporting Information), thereby guiding the homogenization of the Zn^2+^ flux and reducing concentration polarization.

**Figure 6:**
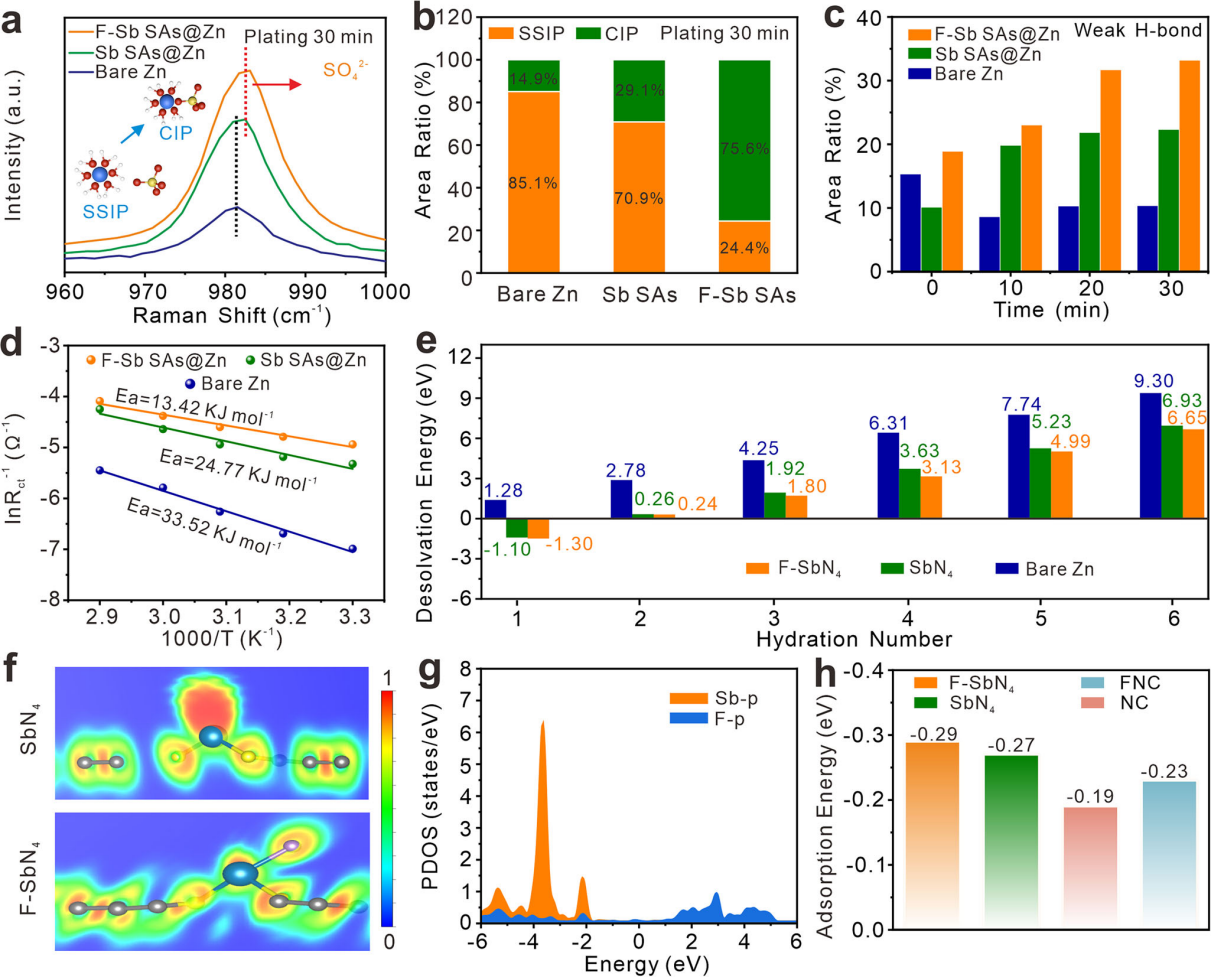
(a) Raman spectra of the ν(SO_4_^2−^) band and b) the SSIP/CIP ratios for different anodes: bare Zn, Sb SAs@Zn, and F‐Sb SAs@Zn. c) In situ Raman spectra of weak hydrogen‐bond proportions evolution during Zn^2+^ deposition. d) Comparison of activation energies (*E*_a_) for bare Zn, Sb SAs@Zn, and F‐Sb SAs@Zn electrodes. e) Comparison of [Zn(H_2_O)_n_]^2+^ (*n*=1–6) desolvation barriers of bare Zn, SbN_4_, and F‐SbN_4_. f) ELF of SbN_4_ and F‐SbN_4_. The blue region represents complete electron delocalization and the red represents complete electron localization. g) The projected density of states (PDOS) of the Sb‐p and F‐p bands. h) Adsorption energy of Zn atom on NC, FNC, SbN_4,_ and F‐SbN_4_.

The role of SAs in regulating the desolvation process is then comprehensively examined. The solvated structure of Zn^2+^ is first analyzed by Raman spectroscopy. The ν(SO_4_^2−^) band in Raman spectra can be deconvoluted into two distinct peaks (Figure S39, Supporting Information), representing solvent separated ion pairs (SSIP, [Zn^2+^(H_2_O)_6_·SO_4_^2−^]) and contact ion pairs (CIP, [Zn^2+^(H_2_O)_5_·OSO_3_^2−^]).^[^ ^53^ ^]^ The higher ratio of CIP indicates promoted desolvation kinetics. In comparison to bare Zn and Sb SAs@Zn, the ν(SO_4_^2−^) band of F‐Sb SAs@Zn shifts to higher wavenumber (**Figure** **6**a), manifesting an elevated proportion of CIP on F‐Sb SAs@Zn. As depicted in Figure 6b, the F‐Sb SAs exhibits the highest CIP ratio of 75.6%, revealing enhanced dehydration of Zn^2+^ solvation shells by F‐Sb SAs. Moreover, in situ Raman spectroscopy is utilized to monitor the configuration of H‐bond network during Zn deposition. Based on Gauss‐Lorenz function, the O‐H stretching vibration of interfacial water can be divided into three characteristic peaks (Figure S40a–c, Supporting Information), corresponding to network water with strong H‐bonds(≈3270 cm^−1^), intermediate water with medium H‐bonds (≈3450 cm^−1^), and free water with weak H‐bonds (≈3550 cm^−1^).^[^ ^54^ ^]^ In aqueous electrolytes, a robust hydrogen bond network stabilizes the [Zn(H_2_O)_6_]^2^⁺ hydration shell, leading to a high desolvation barrier, whereas weak hydrogen bonds destabilize it and lower this barrier. As zinc deposition time prolongs, Sb SAs@Zn and F‐Sb SAs@Zn display diminishing strong H‐bonds ratios, while the bare Zn substrate exhibits an increasing trend in strong H‐bonds (Figure S40d, Supporting Information). The weak H‐bonds proportions of F‐Sb SAs@Zn at all plating time are larger than those of Sb SAs@Zn and bare Zn (Figure 6c), demonstrating that F‐Sb SAs is more favorable to weaken the strong hydrogen bonding network. The higher ratios of weak H‐bonds also imply that more water molecules are removed from the hydrated Zn ions, revealing boosted dehydration kinetics. To further verify the desolvation effect of F‐Sb SAs, the activation energy (*E*_a_) of desolvation is assessed by EIS at different temperatures (Figure S41, Supporting Information). Figure 6d illustrates that the *E*_a_ is greatly diminished from 33.52 to 13.42 kJ mol^−1^ by F‐Sb SAs@Zn, highlighting the capability of F‐Sb SAs in reducing the Zn^2+^ desolvation barrier and improving the Zn^2+^ deposition kinetics.

To elaborate the reason for the accelerated desolvation kinetics on F‐Sb SAs, DFT calculations are performed. The F‐SbN_4_ model costs less energy than SbN_4_ model and bare Zn toward desolvation (Figure 6e). For instance, the F‐SbN_4_ requires merely 6.65 eV to completely release the water molecules from [Zn(H_2_O)_6_]^2+^ to form Zn^2+^, while a high energy of 6.93 eV and 9.30 eV is needed for SbN_4_ and bare Zn, respectively. On the other hand, catalyzed by F‐SbN_4_ and SbN_4_, the bond cleavage between Zn^2+^ and one water molecule becomes thermodynamically spontaneous. We then calculate the electron localization functions (ELF) of F‐SbN_4_ and SbN_4_ to get insight into the physical impact of F‐axial coordination at the electronic level. As illustrated in Figure 6f, the red region surrounding Sb in SbN_4_ indicates electron localization near the Sb center. With the introduction of F‐axial coordination, electron delocalization in the vicinity of Sb atom can be clearly observed. Consequently, the system becomes more incline to accept electrons from Zn, leading to stronger interaction between Zn and F‐SbN_4_. The density of states (DOS) plots in Figure 6g reveal the *p*‐*p* orbital hybridization between Sb and F atoms, implying their strong interaction. As shown in the projected density of states (PDOS), compared to SbN_4_, the *p*‐band center of active Sb site in F‐SbN_4_ gets closer to the Fermi level (Figure S42, Supporting Information). On the other hand, after Zn adsorption (Figure S43, Supporting Information), the energy gap (4.09 eV) between the *d*‐band center of Zn and *p*‐band center of Sb in F‐SbN_4_ system is smaller than that in SbN_4_ system (5.15 eV), promoting orbital hybridization between Zn and Sb atoms and leading to the formation of stronger chemical bonds.^[^ ^55^ ^]^ As a result, the F‐SbN_4_ demonstrates the strongest affinity for Zn with an adsorption energy of −0.29 eV (Figure 6h; Figure S44, Supporting Information), followed by SbN_4_, F,N co‐doped carbon, and N‐doped carbon. The strong desolvation capability of the electrode surface toward [Zn(H_2_O)_6_]^2+^ and its high affinity for Zn^2+^ work sequentially, creating a synergistic effect that collectively promotes uniform Zn deposition. In addition, it is noticed that the adsorption energy of Zn on F,N co‐doped carbon reach ‐0.23 eV. Thus, the integration of F‐axial coordination at Sb site and F‐doped carbon sites within F‐Sb SAs ensuring the best electrochemical activity.

**Figure 7:**
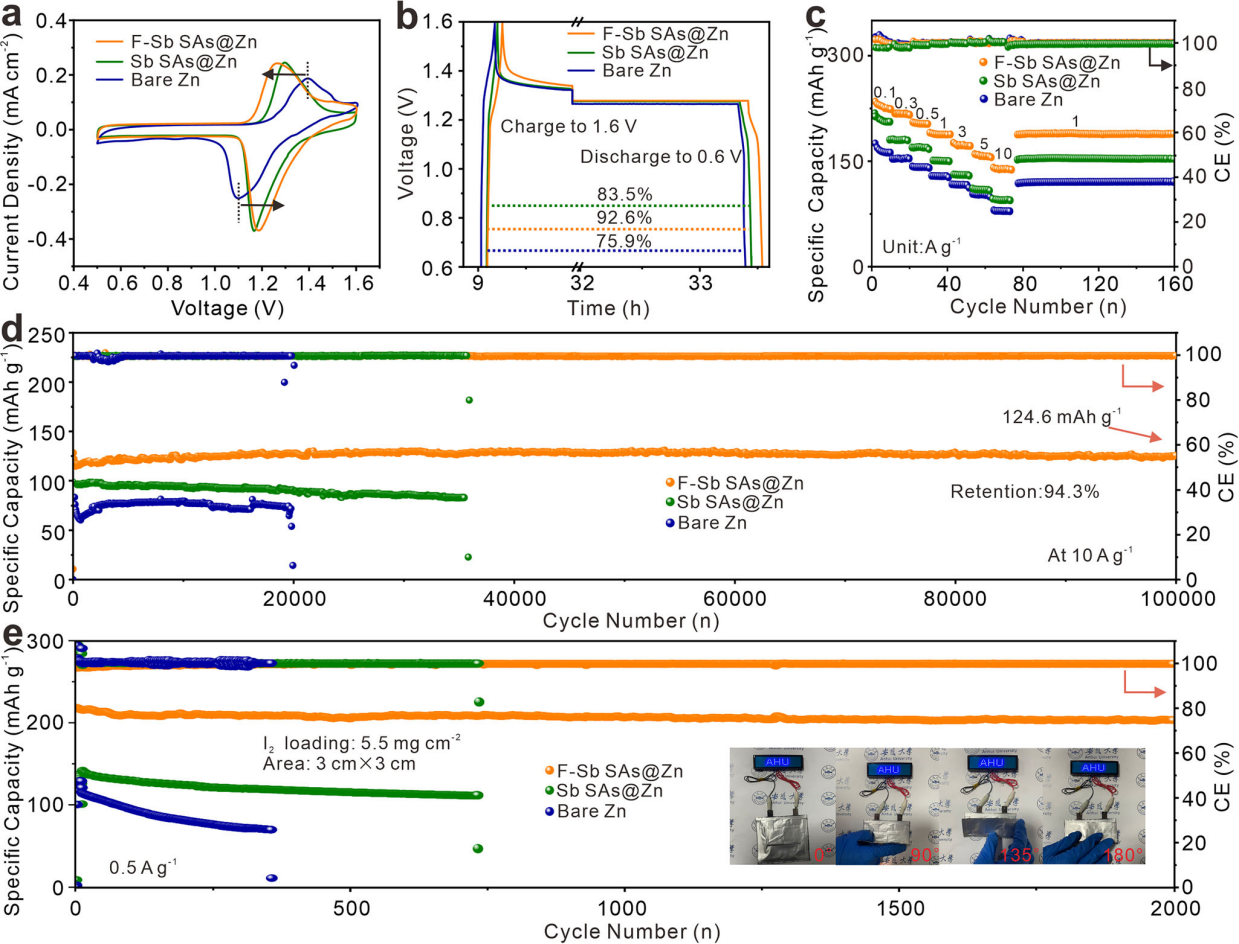
(a) CV curves at a scan rate of 0.5 mV s^−1^, (b) self‐discharging voltage‐time curves and (c) rate performance of Zn||I_2_, Sb SAs@Zn||I_2,_ and F‐Sb SAs@Zn||I_2_ full cells. d) Cycling performance of bare Zn||I_2_, Sb SAs@Zn||I_2_ and F‐Sb SAs@Zn||I_2_ full cells at 10 A g^−1^. e) Cycling performance of pouch cells based on Zn||I_2_, Sb SAs@Zn||I_2_ and F‐Sb SAs@Zn||I_2_ at 0.5 A g^−1^. Inset of panel is the photograph of an LED powered by the F‐Sb SAs@Zn pouch cell.

The functions of F‐Sb SAs are further evaluated in Zn‐I_2_ batteries, with I_2_‐loaded activated carbon (AC) as cathode (Figure S45, Supporting Information). The CV curves of full cells with various anodes manifest that both F‐Sb SAs@Zn||I_2_ and Sb SAs@Zn||I_2_ batteries demonstrate lower oxidation potentials than bare Zn||I_2_ battery (**Figure** **7**a). This is ascribed to the lower charge transfer resistances delivered by F‐Sb SAs and Sb SAs (Figure S46, Supporting Information). Additionally, after 24 h of aging, the F‐Sb SAs@Zn||I_2_ battery shows the highest CE of 92.6% (Figure 7b). The rate performance of the F‐Sb SAs@Zn||I_2_ cell is also superior to that of the Sb SAs@Zn||I_2_ and Zn||I_2_ full cells (Figure 7c). As expected, the F‐Sb SAs@Zn||I_2_ cell exhibits excellent stability. At a high current density of 10 A g^−1^, the F‐Sb SAs@Zn||I_2_ cell can still maintain an outstanding specific capacity of 124.6 mAh g^−1^ even after 100 000 cycles (Figure 7d), with a capacity retention rate of 94.3% and a CE of ≈ 100%. By comparison, the batteries based on bare Zn and Sb SAs@Zn anodes show markedly shorter cycle lifespans.

To demonstrate the practical applicability of the F‐Sb SAs@Zn anode, pouch‐type cells are further fabricated. The F‐Sb SAs@Zn||I_2_ pouch cell achieves excellent cycling performance at 0.5 A g^−1^ (Figure 7e), retaining a high capacity of ≈201 mAh g^−1^ after 2000 cycles, with a capacity retention rate of 92.6%. The F‐Sb SAs@Zn||I_2_ pouch cell can successfully power an LED screen even when subjected to 90°,135°, and 180° bending and recovery, validating impressive flexibility and great feasibility for potential applications. Alongside promoting even Zn^2^⁺ plating and suppressing dendritic growth, the F‐Sb SAs offers robust protection against polyiodide corrosion, securing prolonged cycling stability in zinc‐iodine batteries. Furthermore, the energy density of the pouch cell (61.8 Wh kg^−1^) is comparable to that of most reported works (Table S3, Supporting Information). When the bare Zn, Sb SAs@Zn, and F‐Sb SAs@Zn electrodes are immersed in an I_3_^−^ pregnant ZnSO_4_ electrolyte for 4 h, the in situ UV–vis absorption spectra are recorded. The I_3_^−^ signal on the bare Zn electrode diminishes rapidly (Figure S47a,d, Supporting Information), whereas Sb SAs@Zn displays signal weakening after 30 min (Figure S47b,e, Supporting Information). In stark comparison, the F‐Sb SAs@Zn can maintain a strong and stable I_3_^−^ signal throughout the test (Figure S47c,f, Supporting Information). The surface of F‐Sb SAs@Zn remains smooth and intact (Figure S48, Supporting Information). The improved chemical stability of F‐Sb SAs@Zn can be attributed to the electronegative property of axially coordinated F atom to repel the polyiodide ions and thereby mitigate corrosion reactions. Additionally, the F‐Sb SAs@Zn anode also presents good feasibility in full cell using NH_4_V_4_O_10_ as cathode (Figures S49 and S50, Supporting Information).

## 3 Conclusion

In summary, by using Sb SAs as a paradigm, F‐axial coordination engineering is validated to be an effective approach to improve the desolvation capability and achieve long cycle stability of zinc anodes. The F‐axial coordination can enhance the adsorption of Zn and hinder HER, promote uniform Zn nucleation and deposition while inhibit side reactions. Moreover, the F‐axial coordination is capable of reducing the desolvation activation energy of hydrated Zn^2+^ ions and greatly accelerating the migration kinetics of Zn^2+^ ions. Consequently, the symmetric cell based on F‐Sb SAs exhibits outstanding stability over 6000 h at 5 mA cm^−2^. Furthermore, the F‐Sb SAs@Zn||I_2_ battery demonstrates exceptional cycling stability at a high current density of 10 A g^−1^, achieving a lifespan of 100 000 cycles with a remarkable capacity retention of 94.3%. This work highlights a constructive design strategy at the atomic level to realize highly stable Zn anodes. The coordination regulation strategy developed in this work is also expected to be applicable to other metal single atoms, offering new opportunities for metal anode battery design.

## Conflict of Interest

The authors declare no conflict of interest.

## Supporting information



Supporting Information

## Data Availability

The data that support the findings of this study are available from the corresponding authors upon reasonable request.
