# Supplementary material for: Axial Coordination Induced Electron Delocalization and p‐p Orbital Hybridization in Single‐Atom Catalysts Boosts Zn2+ Desolvation for Highly Stable Zn Anode
Source: Adv Sci (Weinh). 2025 Nov 11;13(4):e18148. doi: 10.1002/advs.202518148 (PMC12822462; doi:10.1002/advs.202518148)
Supplement: Supplementary file 1 — Supporting Information [file ADVS-13-e18148-s001.docx]

**Axial Coordination Induced Electron Delocalization and p-p Orbital Hybridization in Single-Atom Catalysts Boosts Zn^2+^ Desolvation for Highly Stable Zn Anode**

Yan Dao,†^1^ Mengyuan Li,†^1^ Miaomiao Zhang,^1^ Ke Fan,*^1^ Qi Qi,^1,2^ Lin Zhang*^2^ and Xin-Yao Yu*^1^

[1] Y. Dao, M. Li, Q. Qi, Dr. M. Zhang, Dr. K. Fan, Prof. X. Y. Yu
School of Materials Science and Engineering, Anhui University
Hefei 230601, P. R. China
E-mail: yuxinyao@ahu.edu.cn, kefan@ahu.edu.cn

[2] Q. Qi, Prof. L. Zhang

Institute of Solid State Physics, Leibniz University Hannover

Hannover 30167, Germany

E-mail: lin.zhang@fkp.uni-hannover

[†] These two authors contribute equally to this work.

Supporting information for this article is given via a link at the end of the document.

**Experimental Details**

**Materials**

All the chemicals were of analytical reagent grade and used as received without any further treatment. Antimony Trichloride (SbCl_3_), dicyandiamide (DCDA), Trimesic acid was obtained from Macklin Reagent. N-methyl pyrrolidone (NMP, >99.0%) was obtained from Aladdin Reagent. Zinc sulfate (ZnSO_4_) and sulphuric acid (H_2_SO_4_) (GR) were obtained from Sinopharm Chemical Reagent. Polyvinylidene Fluoride (PVDF) was obtained from DoDoChem (Suzhou, China). The ultrapure water was prepared by using deionized water (DIW, 18.25 MΩ cm^−1^).

***Preparation of F-Sb SAs.*** In a typical preparation procedure of F-Sb SAs catalyst, 100 mg antimony chloride (SbCl3), 2.0 g dicyandiamide (DCDA) and 0.2 g Trimesic acid were dissolved in 20 mL of ethanol under ultrasound for 4 h at room temperature. Then, the mixed solution was continuously stirred and dried at 80 ℃. The obtained dried mixture was placed in the porcelain boat and annealed at 800 ℃ under the N2 atmosphere for 2 h with a ramping rate of 5 ℃ min^−1^ to obtain the SAs precursor. The obtained SAs precursor and ammonium fluoride were ground well (weight ratio: 1:10), poured into porcelain boat, and then annealed at 600 ℃ under the N2 atmosphere for 2 h with a ramping rate of 5 ℃ min^−1^. Subsequently, the samples were etched in 1 M H2SO4 solution at 80 ℃ for 24 h to remove the metallic Sb nanoparticles and then washed thoroughly with ethanol and deionized water. Finally, the samples were dried in vacuum at 60 ºC overnight. To fabricate F-Sb SAs-1 and F-Sb SAs-2 with tuned F-incorporated amount, the weight ratios of precursor powder to ammonium fluoride were changed to 1:8 and 1:12, respectively. The layers with thickness of 4.6 μm and 26.3 μm were also prepared and named as F-Sb SAs-3 and F-Sb SAs-4, respectively.

***Preparation of Sb SAs***. To eliminate the effects specific area on the electrochemical performance, the synthetic method was very similar to that of F-Sb SAs. In a typical preparation procedure of Sb SAs catalyst, the SAs precursors (the same to F-Sb SAs) were etched in 1 M H_2_SO_4_ solution at 80 ℃ for 24 h to remove the metallic Sb nanoparticles and then washed thoroughly with ethanol and deionized water.

***Preparation of NC***. The synthesis of NC is similar to that of Sb SAs without the addition of SbCl_3_ and the following etching process.

***Preparation of FNC***. The obtained NC powder and ammonium fluoride were ground well (1:10) and annealed at 600 ℃ under the N_2_ atmosphere for 2 h with a ramping rate of 5 ℃ min^−1^ and the obtained sample is FNC.

***Fabrication of Zn anodes***. Commercial Zn foil (99.99% purity, 0.1 mm) and Ti foil (99.99% purity, 0.01 mm) were cleaned with ethanol before use. The coating materials and polyvinylidene fluoride (PVDF) binder were mixed in NMP solvent in a weight ratio of 8:2 to obtain a slurry. Coating layers were obtained by pouring of the as-obtained slurry onto Zn and Ti foils by means of a squeegee.

***Synthesis of I_2_@AC composite***. The I_2_@AC composite was prepared via an iodine sublimation method. Specifically, 0.5 g of iodine and 0.6 g of activated carbon (AC) were mixed by grinding for 30 minutes. The mixture was then transferred into a Teflon-lined autoclave and heated at 95 °C for 2 hours, followed by an additional 2 h heating at 80 °C to obtain the I₂@AC composite. For the preparation of I₂@AC cathodes, the cathode material (80 wt%), conductive acetylene black (10 wt%), and polytetrafluoroethylene (10 wt%) were mixed, coated onto carbon paper, and dried at 40 °C for 8 h.

***Synthesis of NH_4_V_4_O_10_***. Initially, NH_4_VO_3_ (1.170 g) was dissolved in 35 mL of deionized water at 80°C until complete dissolution. Subsequently, a slow addition of H_2_C_2_O_4_·2H_2_O (1.891g) yielded a dark blue-green solution. This solution was then transferred to a 50 mL autoclave and subjected to heating at 140 °C for 48 h. Afterward, the resulting material underwent washing with deionized water and ethanol, followed by vacuum drying at 80 °C for 12h.

**Materials Characterizations**

X-ray diffraction (XRD, Smartlab) measurements of the electrode and powder samples were performed using Cu *K*_α_ radiation. Scanning electron microscopy (SEM, Zeiss Sigma 500) was employed to observe the morphological differences in the prepared interlayers and to compare the Zn deposition morphology on these electrodes. Raman spectroscopy was conducted using a Renishaw system. X-ray photoelectron spectroscopy (XPS) measurements were performed with a Thermo Fisher Scientific K-Alpha. The internal morphology was characterized by transmission electron microscopy (TEM) and high-resolution TEM (JEM-2100F). HAADF-STEM images were performed with a JEOL JEM-2010 LaB6 operated at 200 kV. XPS data were collected using an ESCALAB250Xi spectrometer with an Al K𝛼 light source. XAFS measurement and data analysis: XAFS spectra at the Sb *L*-edge was collected at BL14W1 station in Shanghai synchrotron radiation facility (SSRF). The Sb *L*-edge XANES data were recorded in a transmission mode. Sb foil and Sb_2_O_3_ were used as references. The elemental content of Sb was analyzed with ICP-OES (iCAP 7400). The deposition behavior of Zn^2+^ on the Zn electrodes was monitored in real time using an YM710TR optical microscope. The UV-Vis spectra (UV-P4, MAPADA) of the soaking solutions were recorded from 200 to 500 nm. The Confocal laser scanning microscopy (CLSM) measurements were performed using a Keyence VK-150K microscope (Japan).

**Electrochemical Characterizations**

The electrochemical performance of bare Zn, NC@Zn, FNC@Zn, F-Sb SAs@Zn and Sb SAs@Zn anodes were evaluated by assembling them into coin-type CR2032 cells. For symmetric cells, Zn anodes were cut into discs with a diameter of 1.2 cm and the electrolyte was a 2 M ZnSO₄ aqueous solution. In the asymmetric configuration, Ti foil served as the counter electrode, while bare Zn, F-Sb SAs@Zn, Sb SAs@Zn, NC@Zn and FNC@Zn electrodes acted as the working electrodes. Full cells were assembled using one of the Zn anodes (bare Zn, F-Sb SAs@Zn, or Sb SAs@Zn) paired with an I₂@AC cathode in 2 M ZnSO₄ electrolyte. Galvanostatic charge/discharge tests were conducted using a NEWARE instrument. Electrochemical impedance spectroscopy (EIS) measurements were performed in the frequency range of 10⁻¹ to 10⁵ Hz with 5 mV amplitude using a Biologic electrochemical workstation (MPG-2). Tafel plots, chronoamperometry (CA), cyclic voltammetry (CV), and linear sweep voltammetry (LSV) were recorded on a CHI 760E electrochemical workstation. Tafel plots for symmetric cells were tested in the voltage range of -0.2 to 0.2 V at a scan rate of 1 mV s^-^¹. CA measurements were conducted on asymmetric cells with a constant overpotential of -150 mV applied for 400 s.

The activation energy (*E*_a_) is calculated according to the Arrhenius equation:

$$\frac{1}{R_{\mathrm{ct}}}\boldsymbol{=}\mathrm{Aexp}\left( \frac{{-E}_{a}}{\mathrm{RT}} \right)$$

Where *R*_ct_ is the charge-transfer resistance, *A* is the pre-exponential factor, *R* is the gas constant, and *T* is the temperature.

The exchange current density is calculated by the Butler-Volmer approximation equation:

$$i=i_{0}\frac{F}{RT}\cdot\frac{\eta}{2}$$

Where *i* is the current density, *i*_0_ is the exchange current density, *η* is the overpotential, *F* is the Faraday constant, *R* is the gas constant, and *T* is the temperature, respectively.

Ionic conductivity tests are carried out on the F-Sb SAs@Ti and bare Ti or stainless steel sheet. The ion conductivity was calculated on the following formula:

$$\delta=\frac{L}{SR_{b}}$$

Where *L* represents the thickness of the coating, *R*_b_ is the resistance according to the EIS measurements. and *S* is the effective area of the layer.

***Adsorption experiments of I_3_⁻.*** The I_3_⁻ solution was synthesized by dissolving I_2_ and KI in deionized water at a molar ration of 1:100. Subsequently, F-Sb SAs@Zn, Sb SAs@Zn, and bare Zn were added to or immersed in this solution to observe their adsorption abilities for soluble iodine species.

**COMSOL Calculations**

The electric field distribution and the concentration of Zn ions on the anode surface were simulated by finite element analysis using the chemical dilute matter transfer and electrostatic field coupling of COMSOL Multiphysics 6.2. Based on the difference of Zn anode surface morphology, we constructed different physical models. The simulation area is 5μm×5μm, the upper boundary of the model is the cathode, the lower boundary is the anode, and the two sides are insulating boundaries. The initial potential between the two electrodes in the model is 0.2 V, the Zn^2+^ concentration is 2 M, and the current density is 1 mA cm^-2^, in which the diffusion of zinc ions in the electrolyte follows Fick's law with a diffusion coefficient of 3.3 × 10^-10^ m^2^/s and the electromobility follows the Nernst-Einstein relationship. In order to ensure the accuracy of the calculation results, a free triangular mesh was used to mesh the model and the MUMPS (Multifrontal Massively Parallel Solver) direct solver was used to focus on the simulation of the first 5 seconds variation of the battery charging process.

**DFT Computational Methods**

First-principles calculations were performed by VASP in the framework of DFT^[1]^. The exchange-correlation energy was described by the GGA with the PBE flavor^[2]^. The van der Waals interaction between adatoms and substrates were considered by using DFT-D2^[3]^. approach. To avoid the effect of interlayer interaction, a vacuum layer of 15 Å along the *c*-axis was added. For structural optimization, a plane-wave cutoff energy of 400 eV was adopted for the following systems: 6×5×1 supercells of SbN_4_, F-SbN_4_, N-doped carbon, and F,N co-doped carbon, a 3×3×1 supercell of Sb and the 3×3×1 supercell of Zn (002)^[4]^. A 3×3×1 *k*-point mesh was used for all five systems. The energy and force convergence thresholds were set to 10⁻^5^ eV and 0.02 eV Å⁻^1^, respectively. The adsorption energy (*E*_ad_) and Gibbs free energy change (Δ*G*, T=298 K) were calculated by the following equations:

$$E_{ad} =E_{\left[ substrate+adatom \right]}-E_{\left[ substrate \right]}-E_{\left[ adatom \right]}$$

$$\Delta G=E_{ad}+\Delta E_{ZPE}-T\Delta S$$

where *E*_ad_, Δ*E*_ZPE_, and *T*Δ*S* represent the changes of DFT-calculated total energy with the adatoms, zero-point energy (ZPE), and entropic contribution, respectively.

**Statistical Analysis**

All experimental data were derived from at least three independent replicates (n ≥ 3) unless otherwise specified. The LSV measurements in the three-electrode system were performed using the CHI760E electrochemical workstation software with 90% i*R* compensation applied. All data testing of coin cells and pouch cells were obtained from the NEWARE system and the EC-Lab electrochemical workstation.


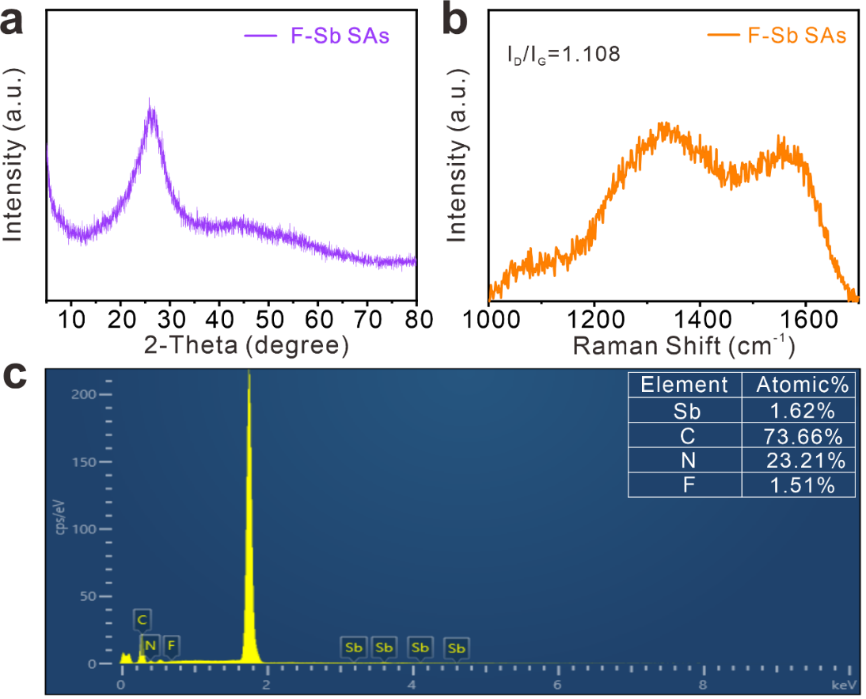


**Figure S1.** (a) XRD pattern, (b) Raman spectrum, and (c) EDS spectrum of F-Sb SAs.


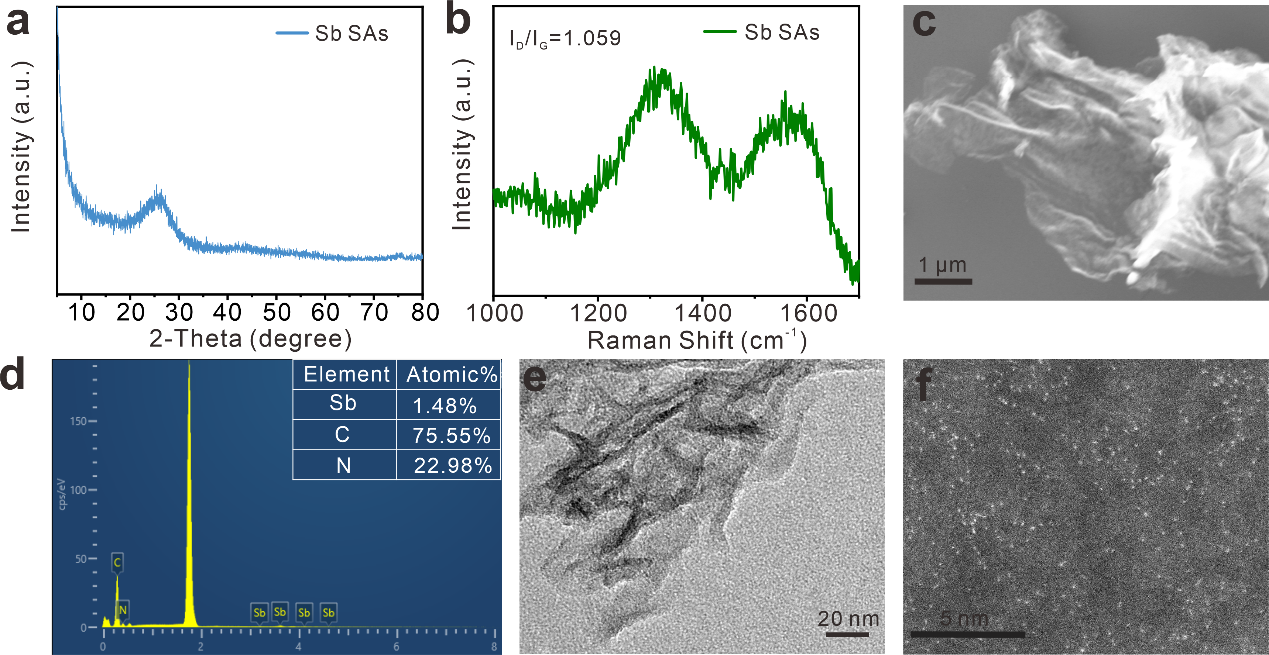


**Figure S2.** (a) XRD pattern, (b) Raman spectra, (c) SEM image, (d) EDS spectrum, (e) TEM image, and (f) magnified HAADF-STEM image of Sb SAs.


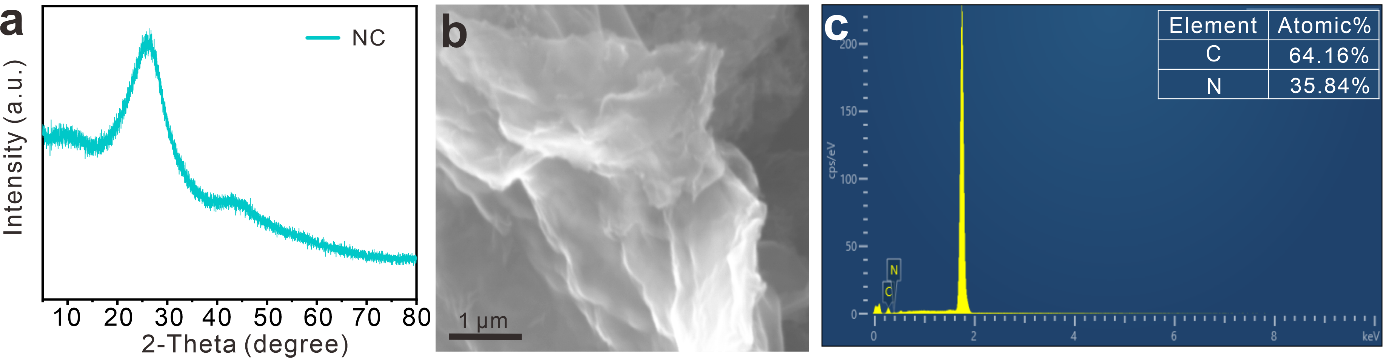


**Figure S3.** (a) XRD pattern, (b) SEM image and (c) EDS spectrum of NC.


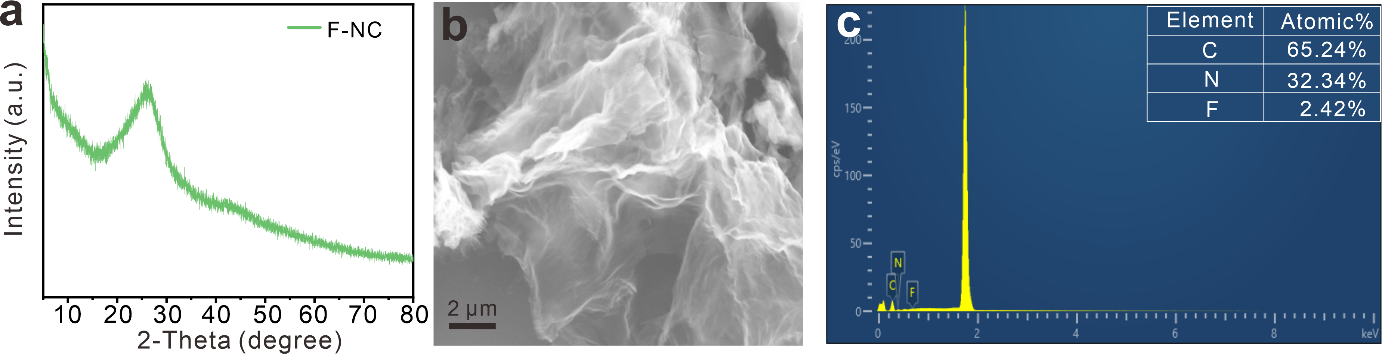


**Figure S4.** (a) XRD pattern, (b) SEM image and (c) EDS spectrum of FNC.


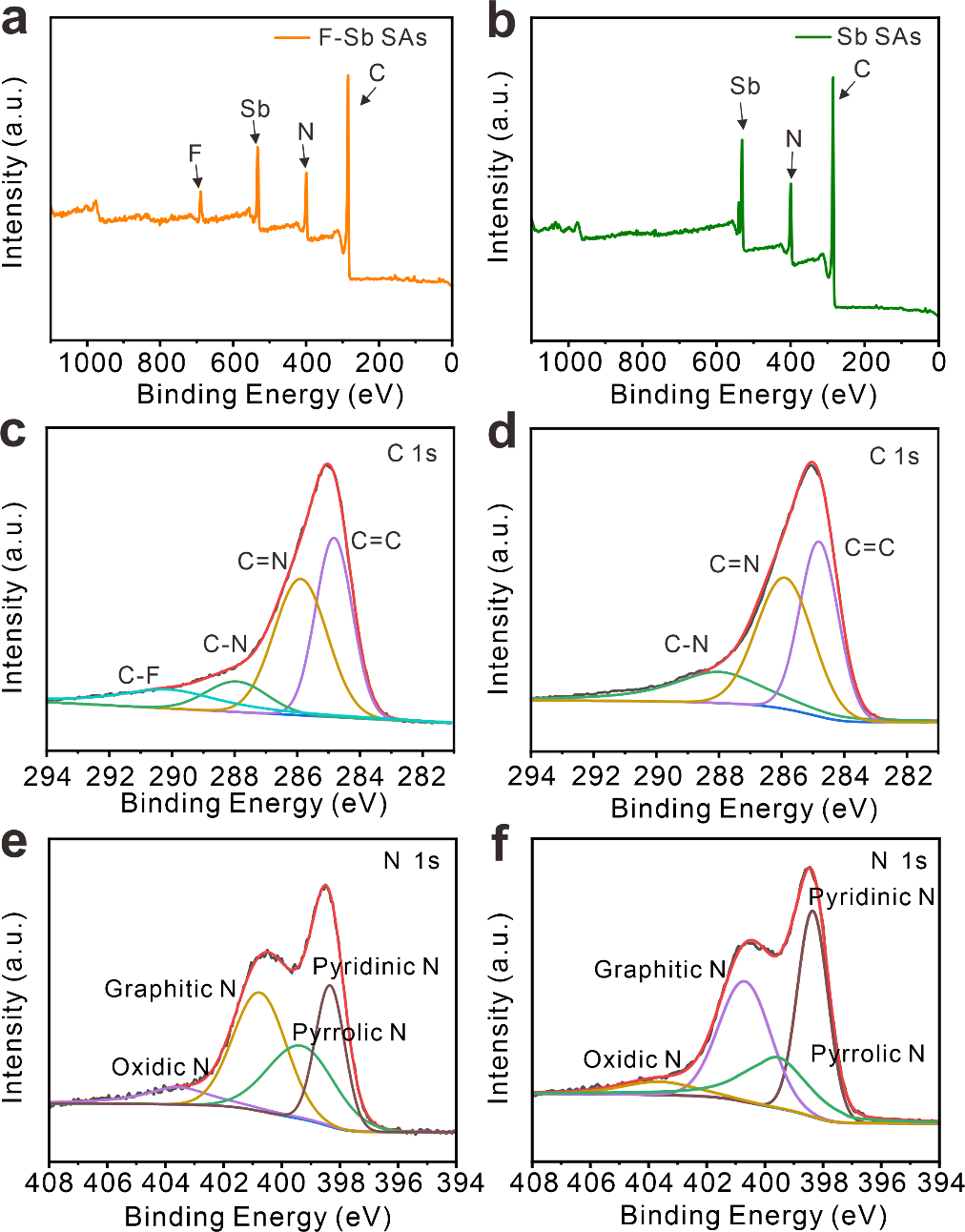


**Figure S5**. (a,b) Survey XPS spectra of F-Sb SAs and Sb SAs; High-resolution C 1s XPS spectra of F-Sb SAs (c) and Sb SAs (d). High-resolution N 1s XPS spectra of F-Sb SAs (e) and Sb SAs (f).


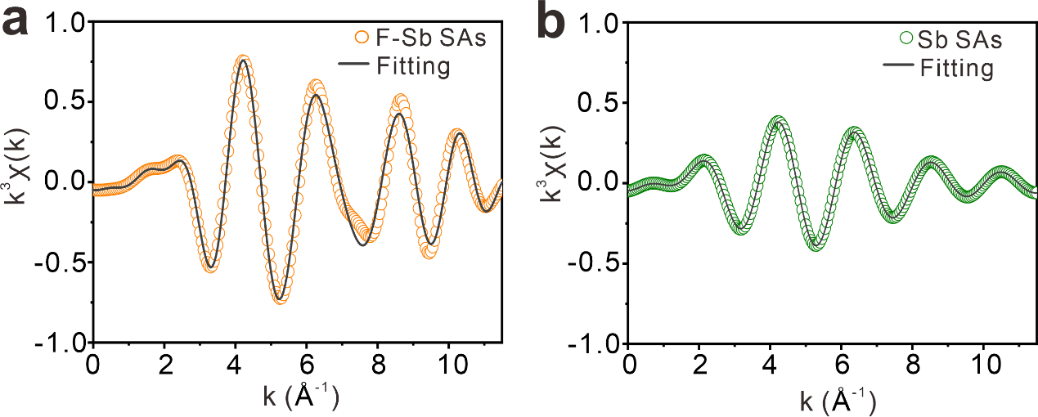


**Figure S6**. FT-EXAFS fitting for Sb *k* space curves of (a) F-Sb SAs and (b) Sb SAs.


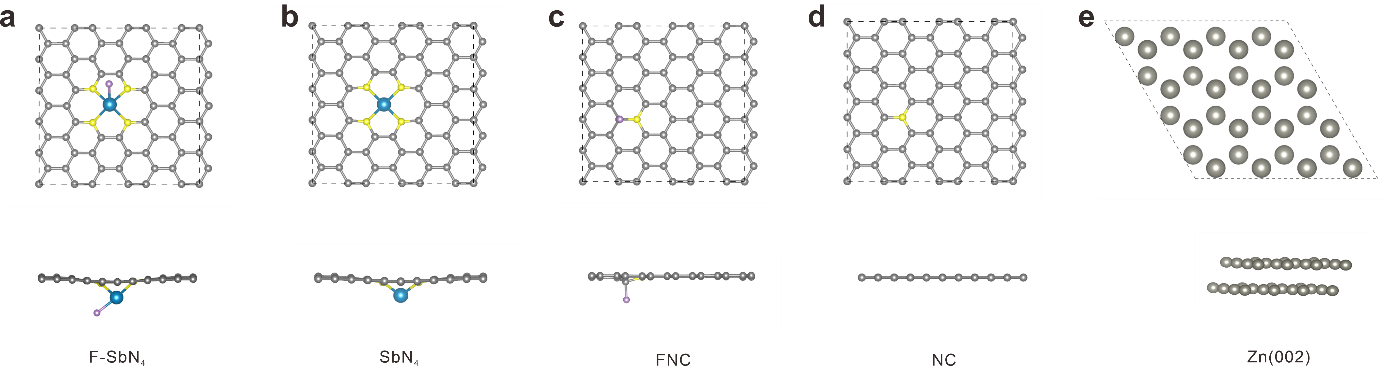


**Figure S7**. The top view of the optimized structure of (a) F-SbN_4_, (b) SbN_4_, (c) FNC, (d) NC and (e) Zn (002). The grey, blue, yellow, purple and dark gray balls represent C, Sb, N, F and Zn atoms, respectively.


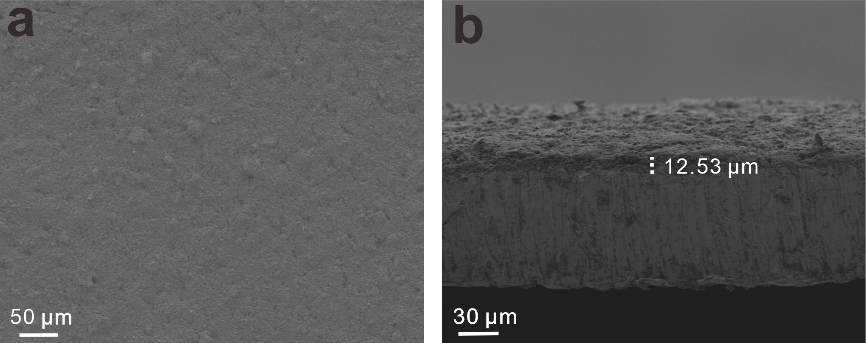


**Figure S8**. (a) Top view SEM image and (b) cross-section SEM image of F-Sb SAs layer on Zn foil.


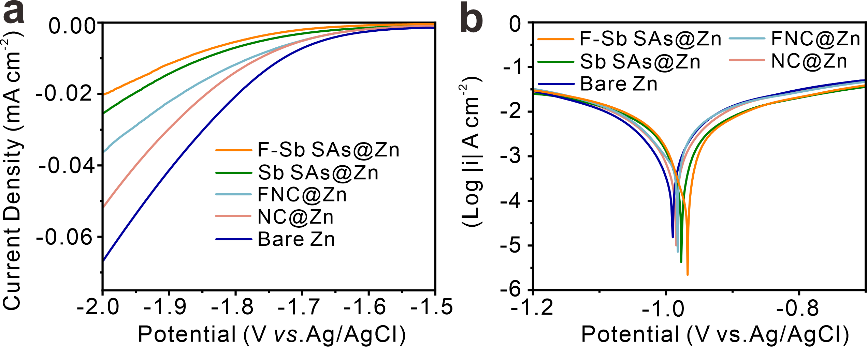


**Figure S9**. (a) LSV curves and (b) Tafel curves of bare Zn, NC@Zn, FNC@Zn, Sb SAs@Zn and F-Sb SAs@Zn tested using three-electrode system. LSV curves (in 1 M Na₂SO₄) and Tafel curves (in 2 M ZnSO₄) in a three-electrode system with a Zn anode (WE), Pt (CE), and Ag/AgCl (RE). Scan rate: 10 mV s⁻¹.


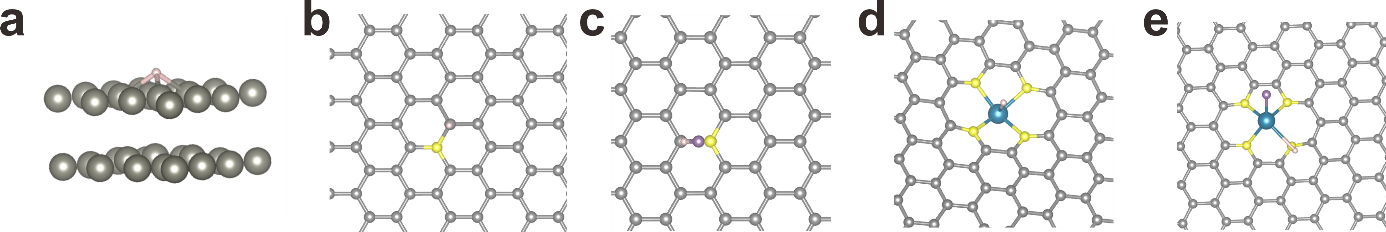


**Figure S10.** The view of the optimized structure of (a) Zn (002)-H, (b) NC-H, (c) FNC-H, (d) SbN_4_-H and (e) F-SbN_4_-H. The pink ball represents H atom.


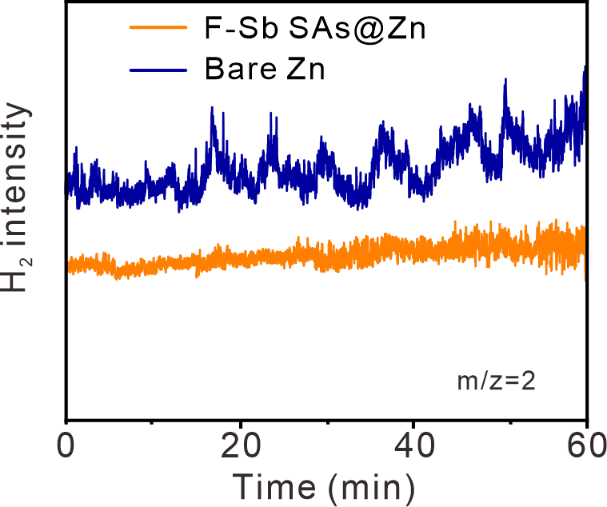


**Figure S11.** DEMS tests monitoring H_2_ emissions of Zn plating/stripping processes of bare Zn and F-Sb SAs@Zn.


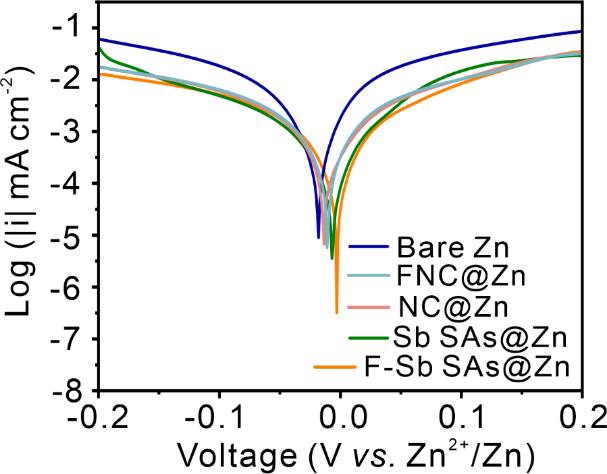


**Figure S12**. Tafel curves of bare Zn, NC@Zn, FNC@Zn, Sb SAs@Zn and F-Sb SAs@Zn.


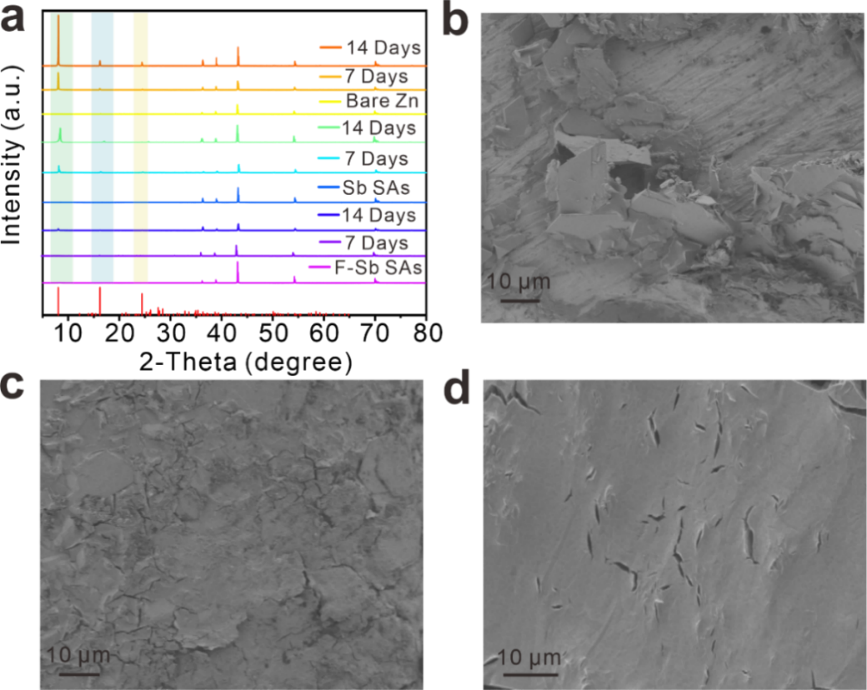


**Figure S13**. (a) XRD patterns of bare Zn, Sb SAs@Zn and F-Sb SAs@Zn anodes after 7 days and 14 days immersion in 2 M ZnSO₄ electrolyte. Corresponding SEM images of (b) bare Zn, (c) Sb SAs@Zn and (d) F-Sb SAs@Zn after 14 days of immersion.


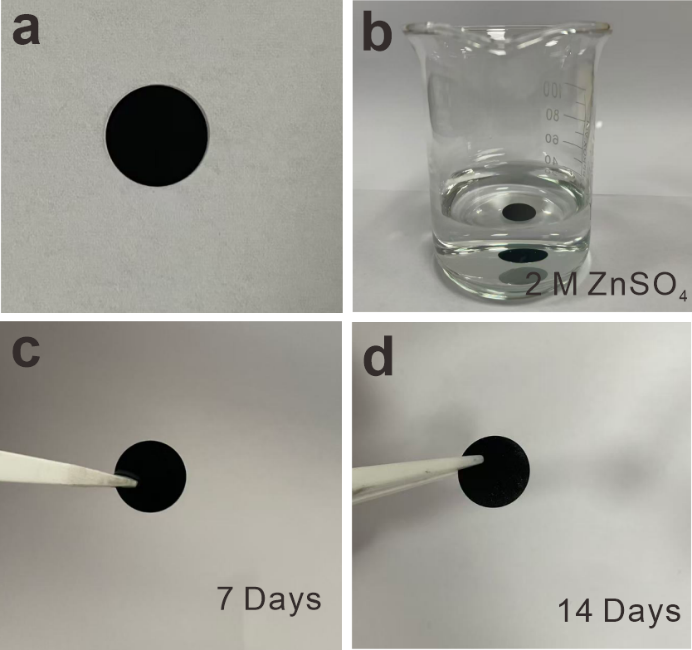


**Figure S14**. The digital images of F-Sb SAs@Zn anode before immersion (a), immersion in 2 M ZnSO₄ electrolyte (b), immersion after 7 days (c), and immersion after 14 days (d).


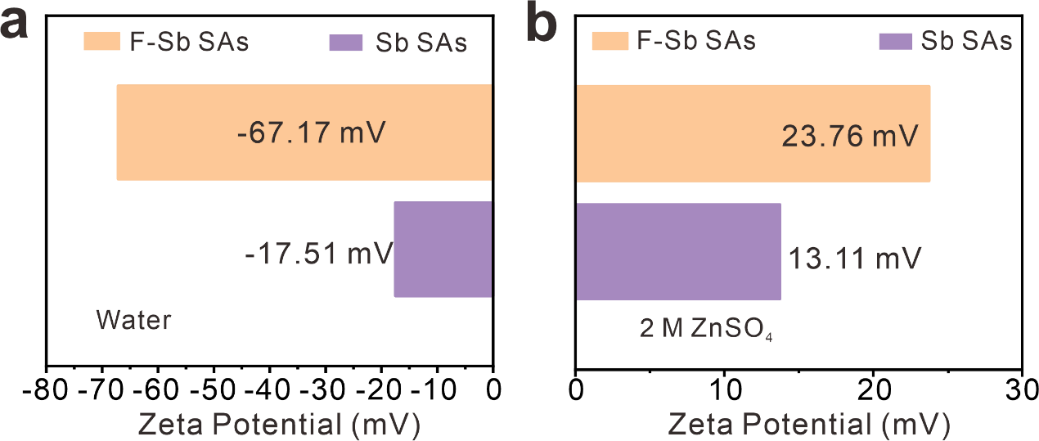


**Figure S15**. Zeta potentials of F-Sb SAs and Sb SAs in (a) water and (b) 2 M ZnSO_4_.


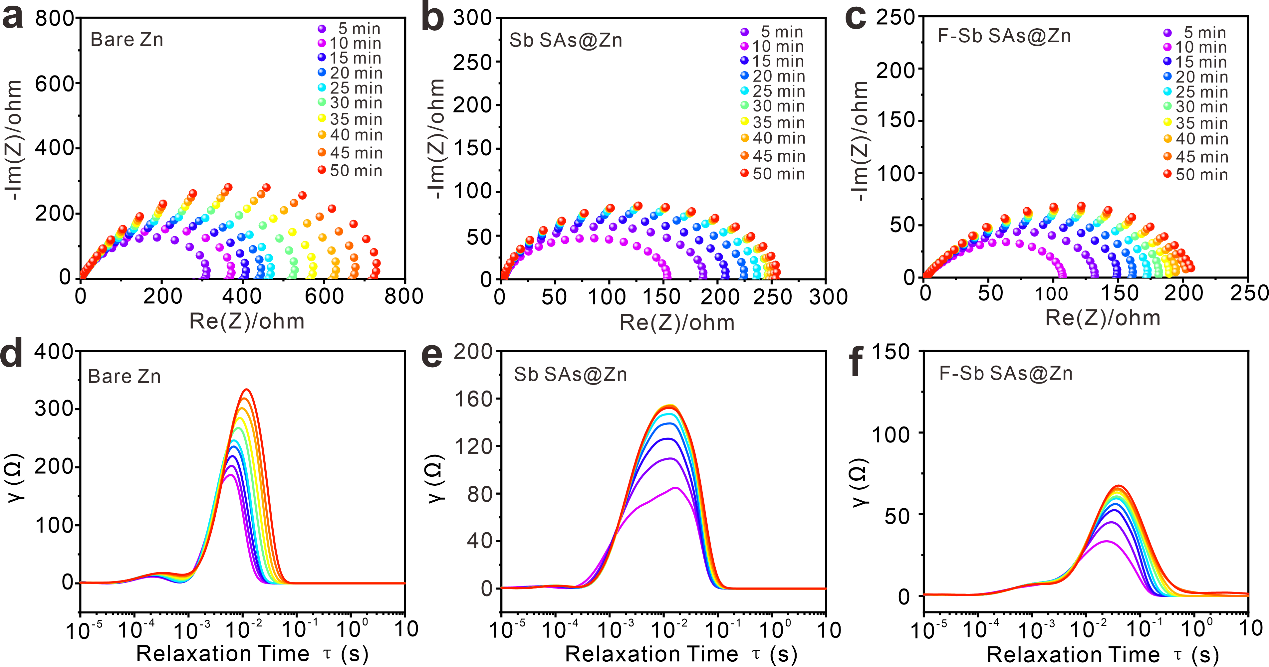


**Figure S16**. Operando EIS evolution of symmetric cells based on (a,d) bare Zn, (b,e) Sb SAs@Zn and (c,f) F-Sb SAs@Zn. The EIS measurements were performed after cells rested for 5 min.


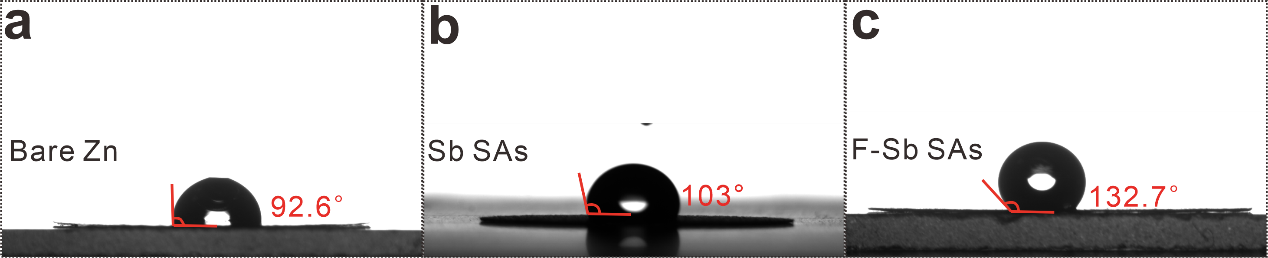


**Figure S17**. The contact angles of (a) bare Zn, (b) Sb SAs@Zn and (c) F-Sb SAs@Zn.


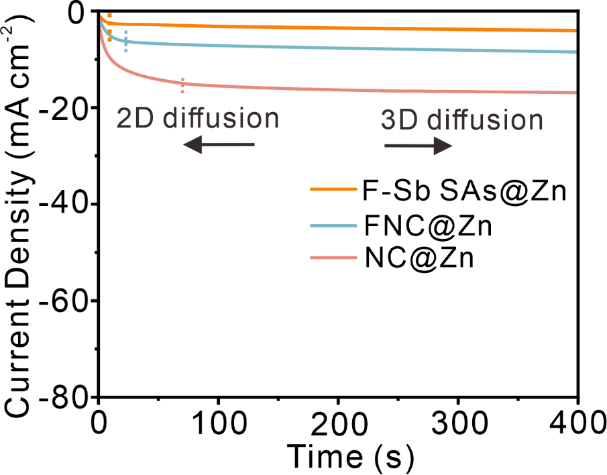


**Figure S18**. CA curves of FNC@Zn, NC@Zn and F-Sb SAs@Zn.


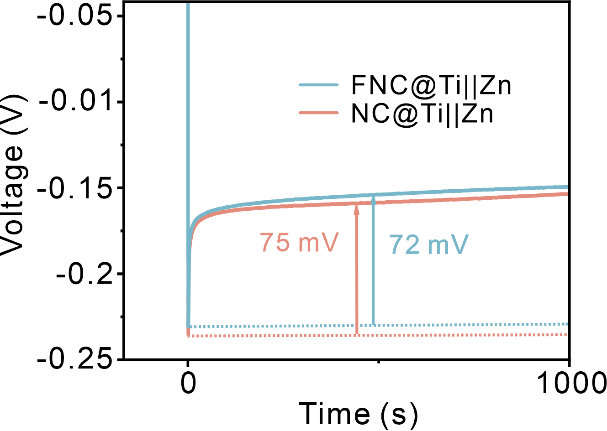


**Figure S19.** Voltage-time curves of Zn plating on FNC@Ti||Zn and NC@Ti||Zn at 2 mA cm^-2^.


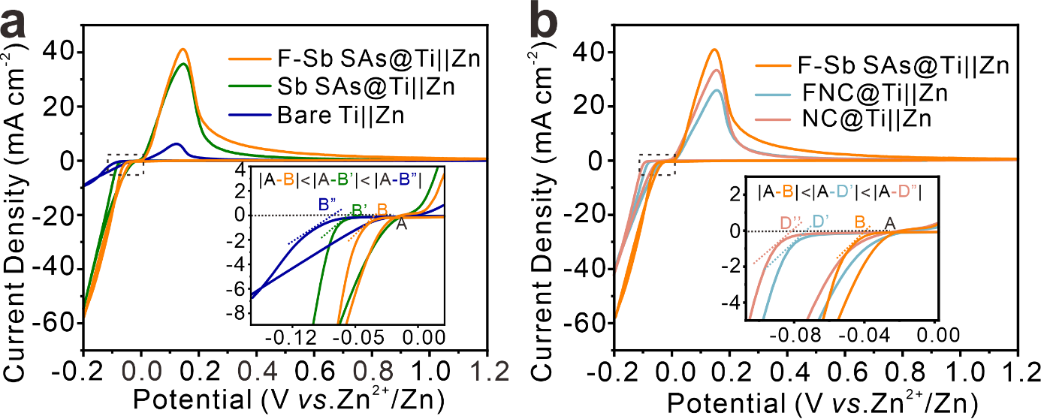


**Figure S20**. CV curves of bare Ti||Zn, FNC@Ti||Zn, NC@Ti||Zn, Sb SAs@Ti||Zn and F-Sb SAs@Ti||Zn.


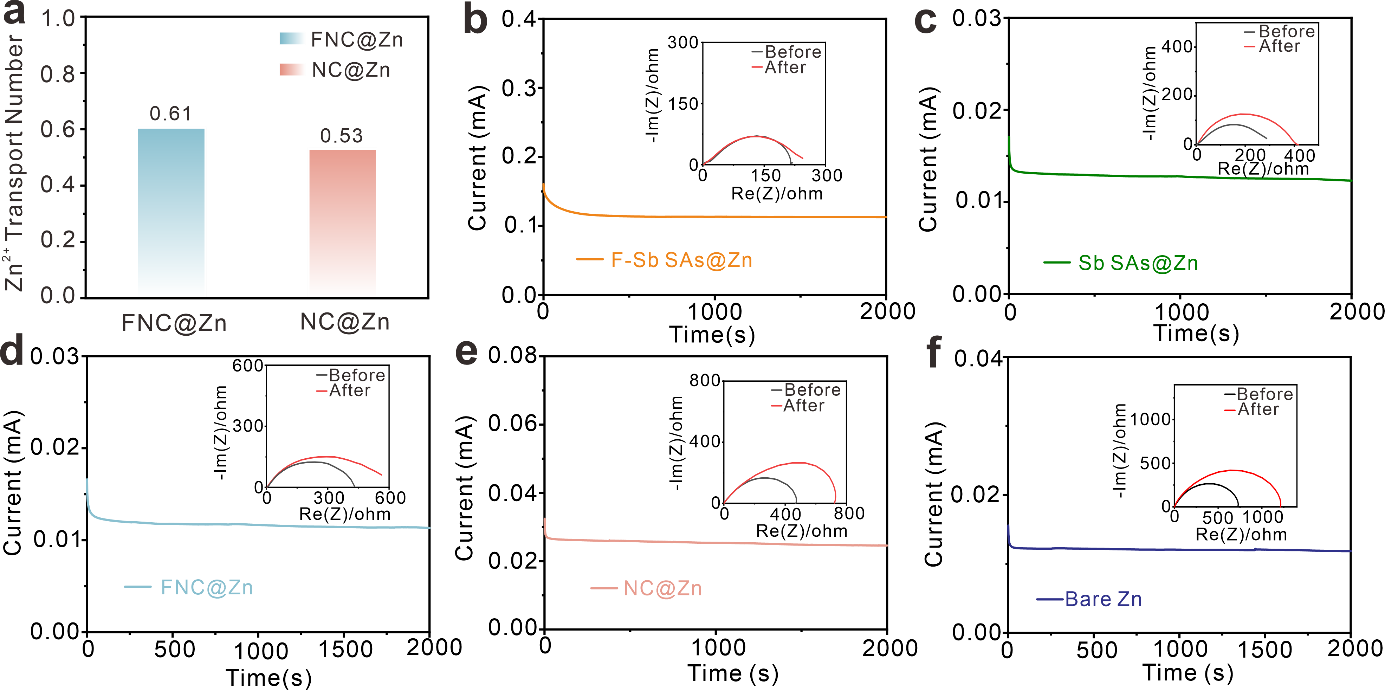


**Figure S21.** (a) Comparison of Zn^2+^ transport number of FNC@Zn and NC@Zn. EIS curves of the symmetric cells for (b) F-Sb SAs@Zn, (c) Sb SAs@Zn, (d) FNC@Zn, (e) NC@Zn and (f) bare Zn before and after polarization. The insets are CA curves polarized at constant voltage of 25 mV for 2000 s.


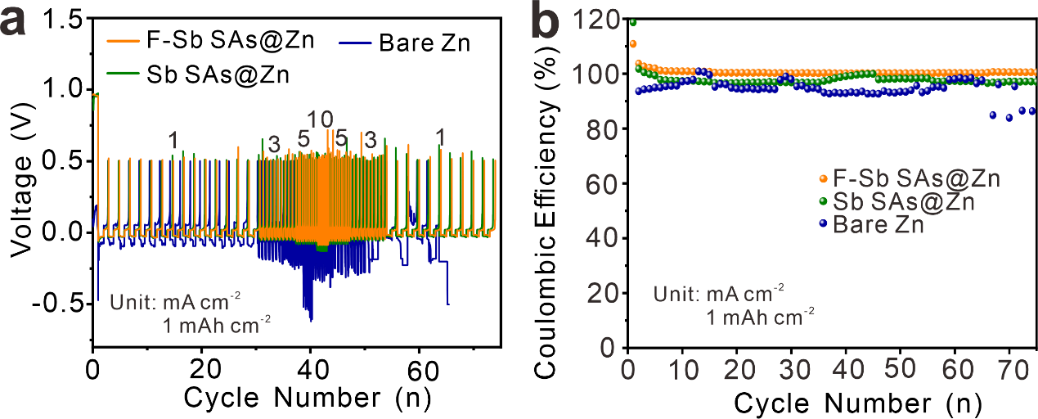


**Figure S22**. (a) Comparison of voltage profiles and (b) CE profiles of the asymmetric Zn||Ti, Sb SAs@Zn||Ti and F-Sb SAs@Zn||Ti cells at the various current densities ranging from 1 to 10 mA cm^-2^.


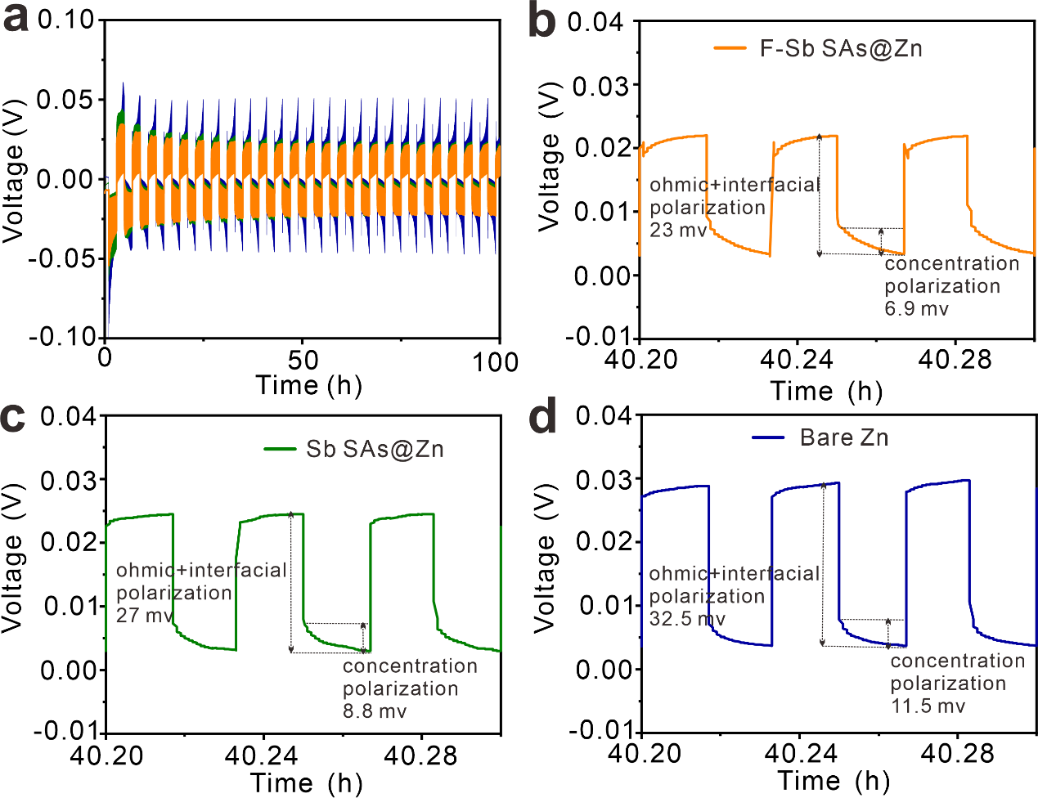


**Figure S23**. (a) GITT curves of bare Zn, Sb SAs@Zn, and F-Sb SAs@Zn. The partial enlargement of GITT curves for (b) F-Sb SAs@Zn, (c) Sb SAs@Zn and (d) bare Zn.


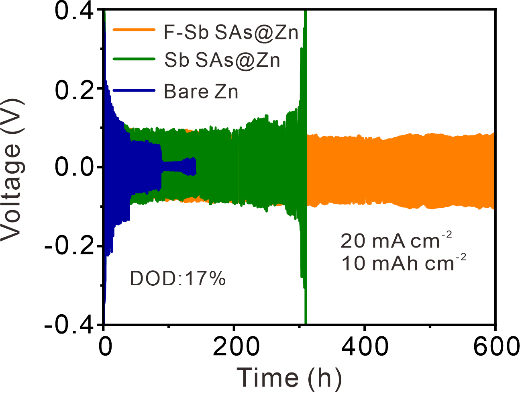


**Figure S24**. Cycling stability at 20 mA cm^−2^ and 10 mAh cm^−2^.


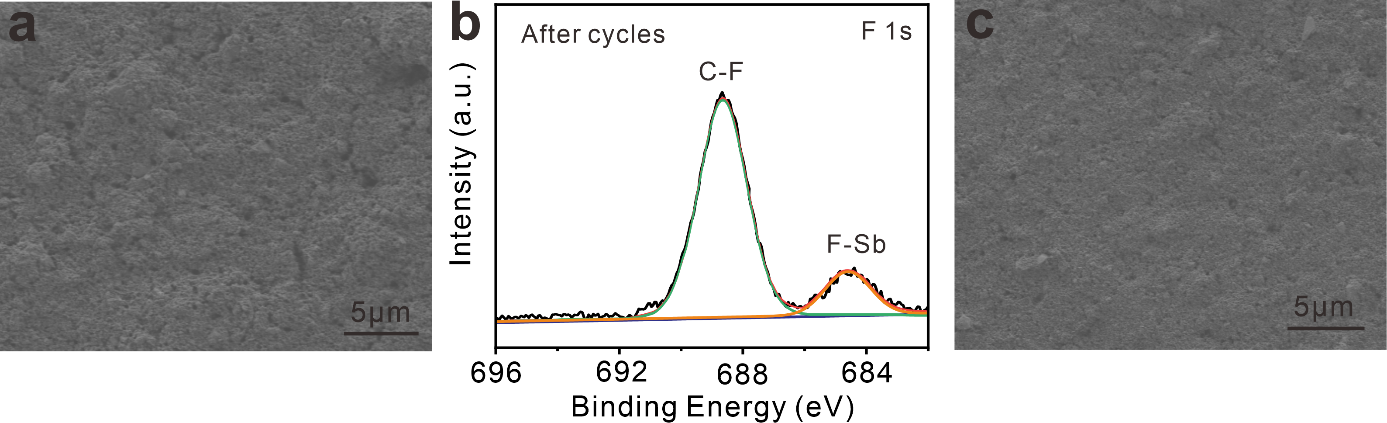


**Figure S25.** (a) SEM image of the F-Sb SAs@Zn before cycling. (b) F 1s XPS spectrum and (c) SEM image of the F-Sb SAs@Zn after cycling at 5 mA cm^-2^ and 1 mAh cm^-2^.


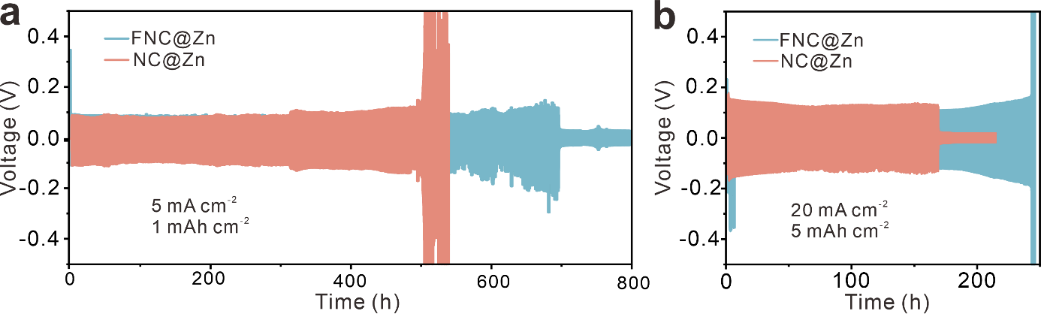


**Figure S26.** (a) Cycling stability at 5 mA cm^-2^ and 1 mAh cm^-2^ and (b) Cycling stability at 20 mA cm^-2^ and 5 mAh cm^-2^ of FNC@Zn and NC@Zn symmetric cells.


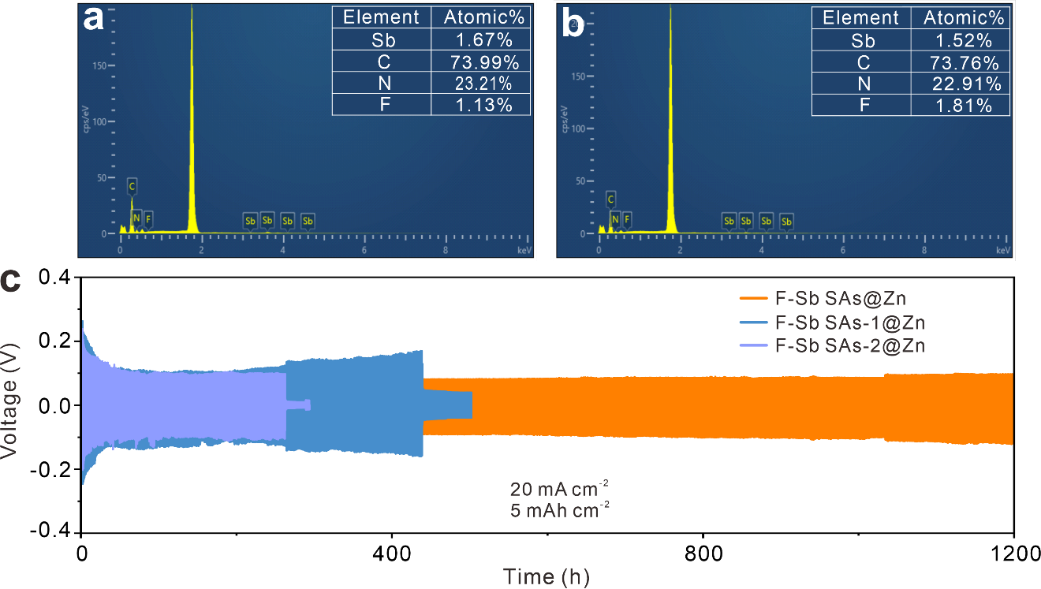


**Figure S27**. EDS spectra of F-Sb SAs-1 (a) and F-Sb SAs-2 (b). (c) Cycling stability of F-Sb SAs, F-Sb SAs-1, and F-Sb SAs-2.


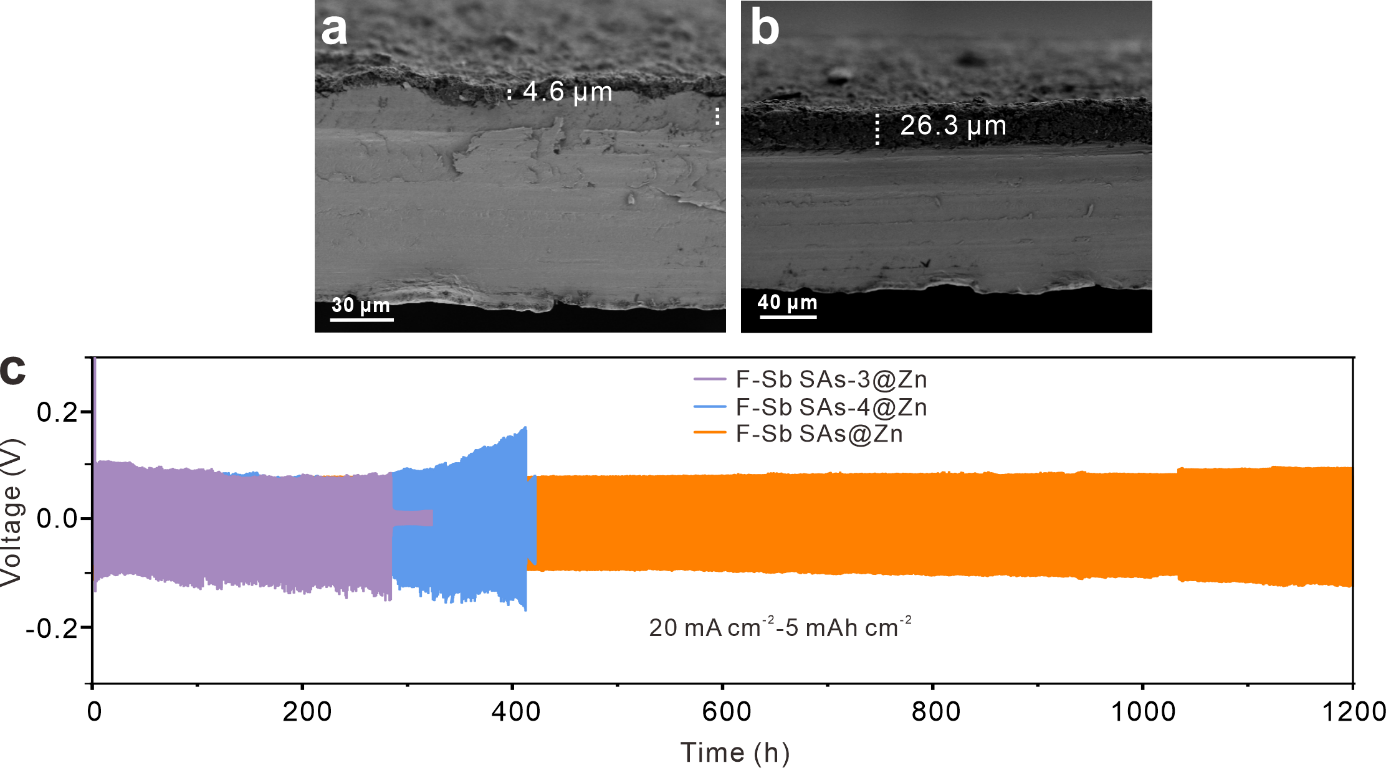


**Figure S28**. The SEM coating thickness of (a) F-Sb SAs-3@Zn, (b) F-Sb SAs-4@Zn. (c) Cycling stability of F-Sb SAs, F-Sb SAs-3, and F-Sb SAs-4.


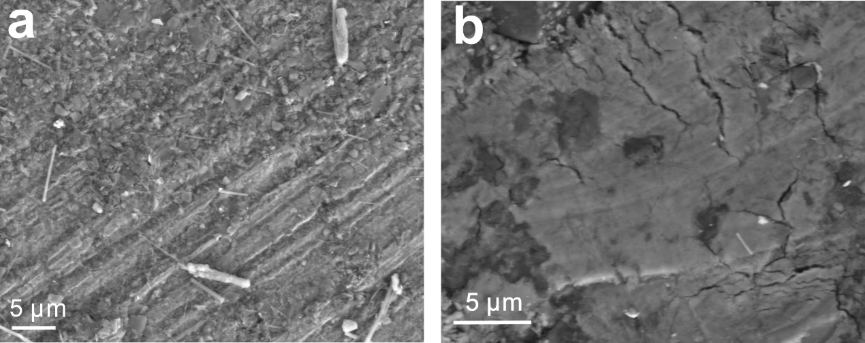


**Figure S29**. Top view SEM images of (a) bare Zn and (b) F-Sb SAs@Zn anodes after plating at 5 mA cm^-2^ and 1 mAh cm^-2^.


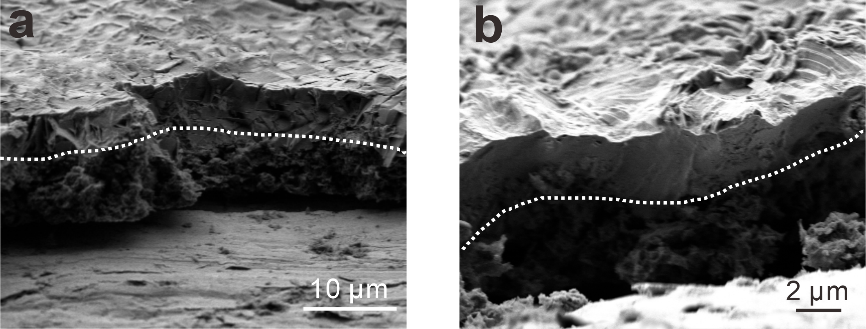


**Figure S30.** The Cross-section SEM images of zinc deposition on F-Sb SAs@Zn.


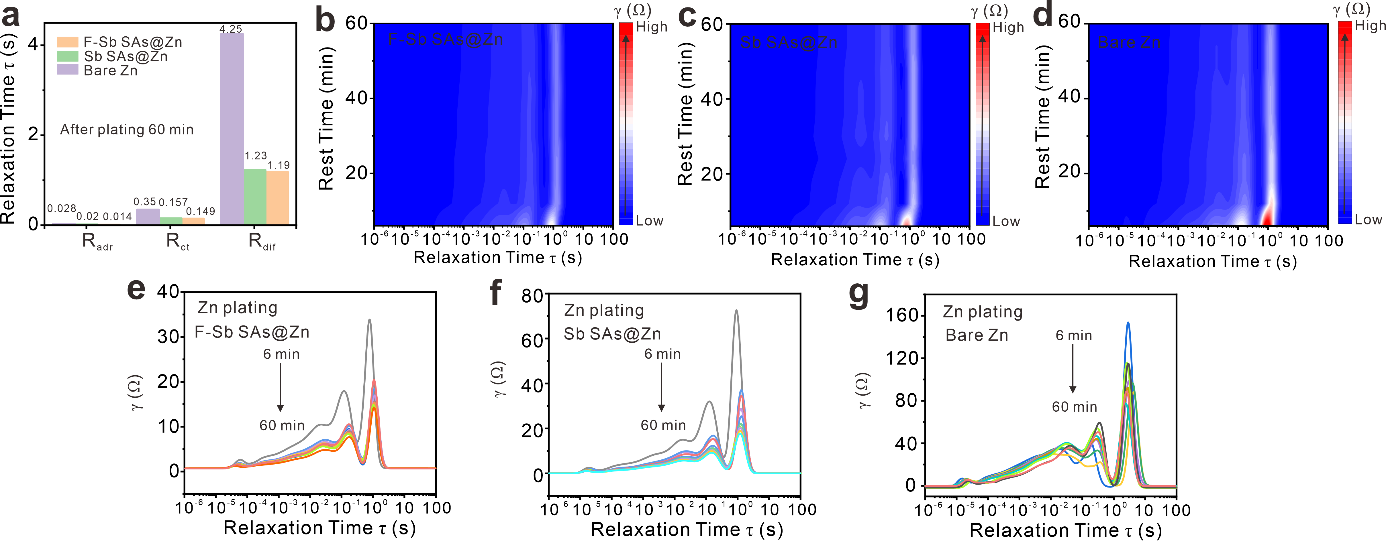


**Figure S31.** (a) The comparation of relaxation time (τ) changes of diverse interfacial process after plating 60 min. DRT curves of F-Sb SAs@Zn (b,e), Sb SAs@Zn (c,f) and Bare Zn (d, g) during Zn deposition at 1 mA cm^-2^ for 3 min and rested for 3 min.


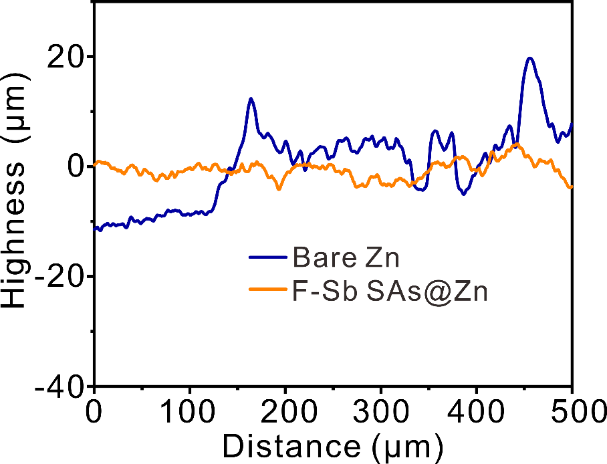


**Figure S32**. 3D CLSM corresponding surface roughness profiles of bare Zn and F-Sb SAs@Zn electrodes after 100 cycles at 5 mA cm^-2^ and 1 mAh cm^-2^.


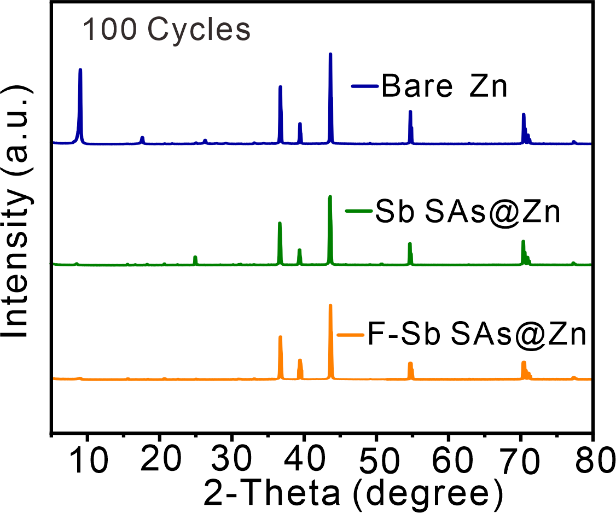


**Figure S33**. XRD patterns of bare Zn, Sb SAs@Zn and F-Sb SAs@Zn anodes after 100 cycles at 5 mA cm^-2^ and 1 mAh cm^-2^.


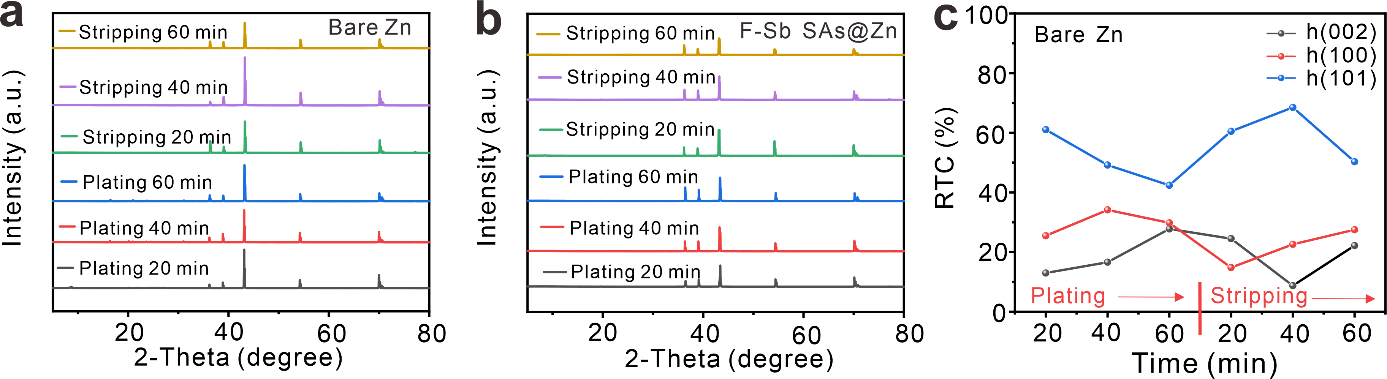


**Figure S34**. XRD patterns during Zn plating/stripping at 5 mA cm^−2^: (a) bare Zn and (b) F-Sb SAs@Zn. Line charts of fitted RTCs for (002), (100) and (101) Zn planes of (c) bare Zn.


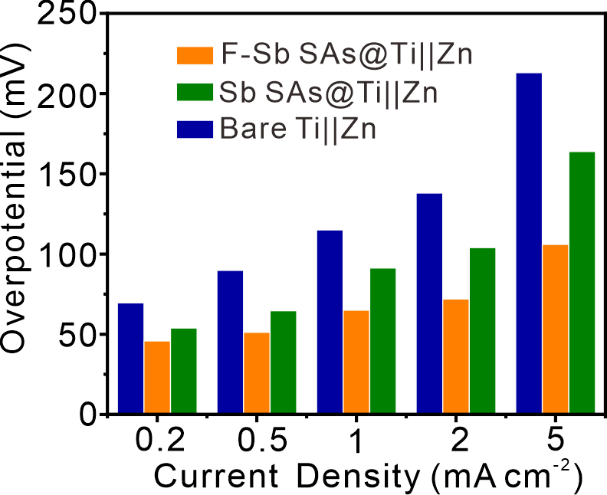


**Figure S35**. Nucleation overpotential plots at different current densities.


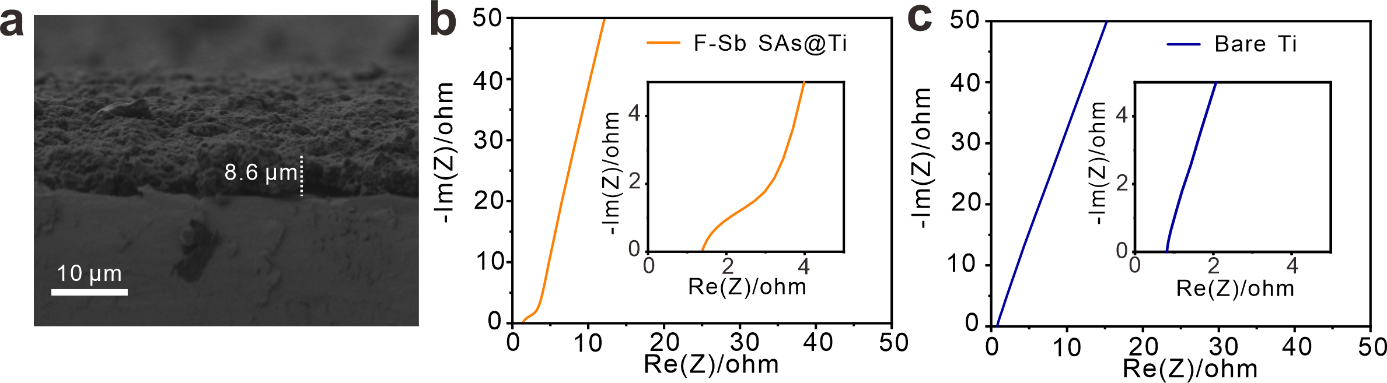


**Figure S36**. (a) Cross-section SEM image of F-Sb SAs layer. EIS plots of (b) F-Sb SAs@Ti||F-Sb SAs@Ti and (c) Bare Ti||bare Ti symmetrical cells. The inset is the enlargement of the data in the range of 0~5 Ω. The ionic conductivity of F-Sb SAs is 15.51×10^−4^ S cm^−1^.


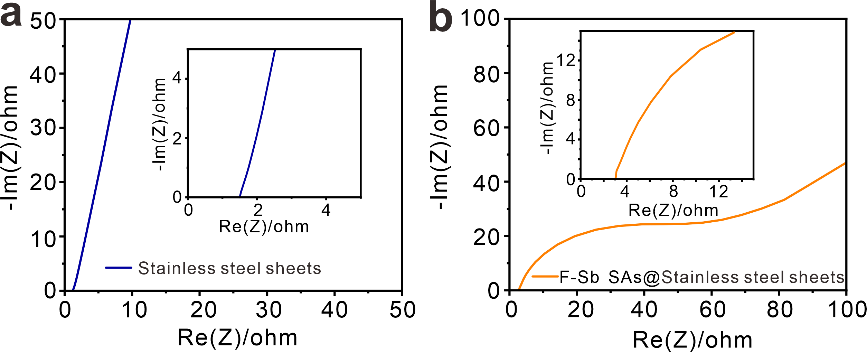


**Figure S37.** EIS plots of (a) Stainless steel sheets||stainless steel sheets and (b) F-Sb SAs@stainless steel sheets||F-Sb SAs@stainless steel sheets symmetrical cells. The inset is the enlargement of the data. The ionic conductivity of F-Sb SAs is 11.49×10^−4^ S cm^−1^.


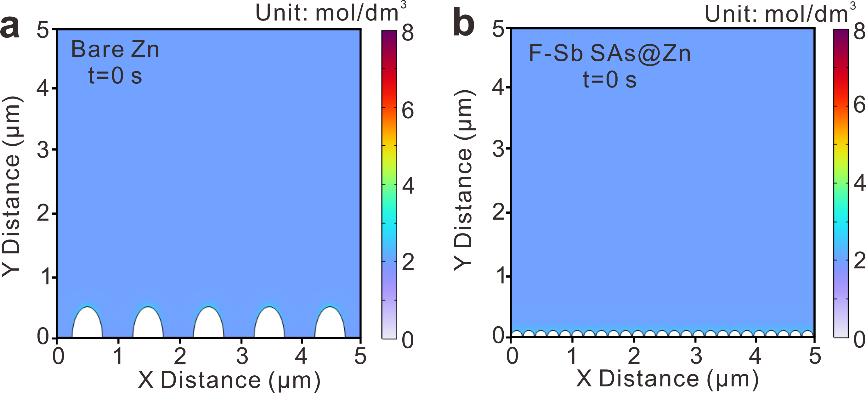


**Figure S38**. The dynamic COMSOL FEM simulation of Zn^2+^ concentration (*t* = 0 s) for (a) bare Zn electrode and (b) F-Sb SAs@Zn electrode.


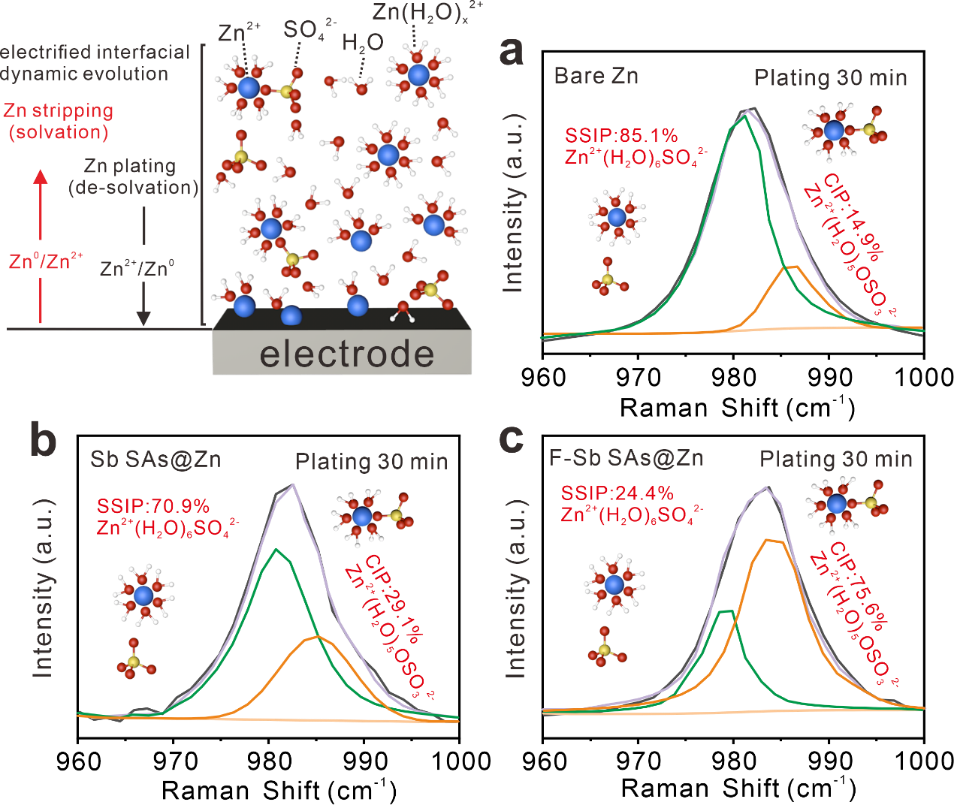


**Figure S39.** (a) Raman spectra of the 𝜈(SO_4_^2-^) band and the SSIP/CIP ratios for different anodes: (b) bare Zn, (c) Sb SAs@Zn and (d) F-Sb SAs@Zn.

**
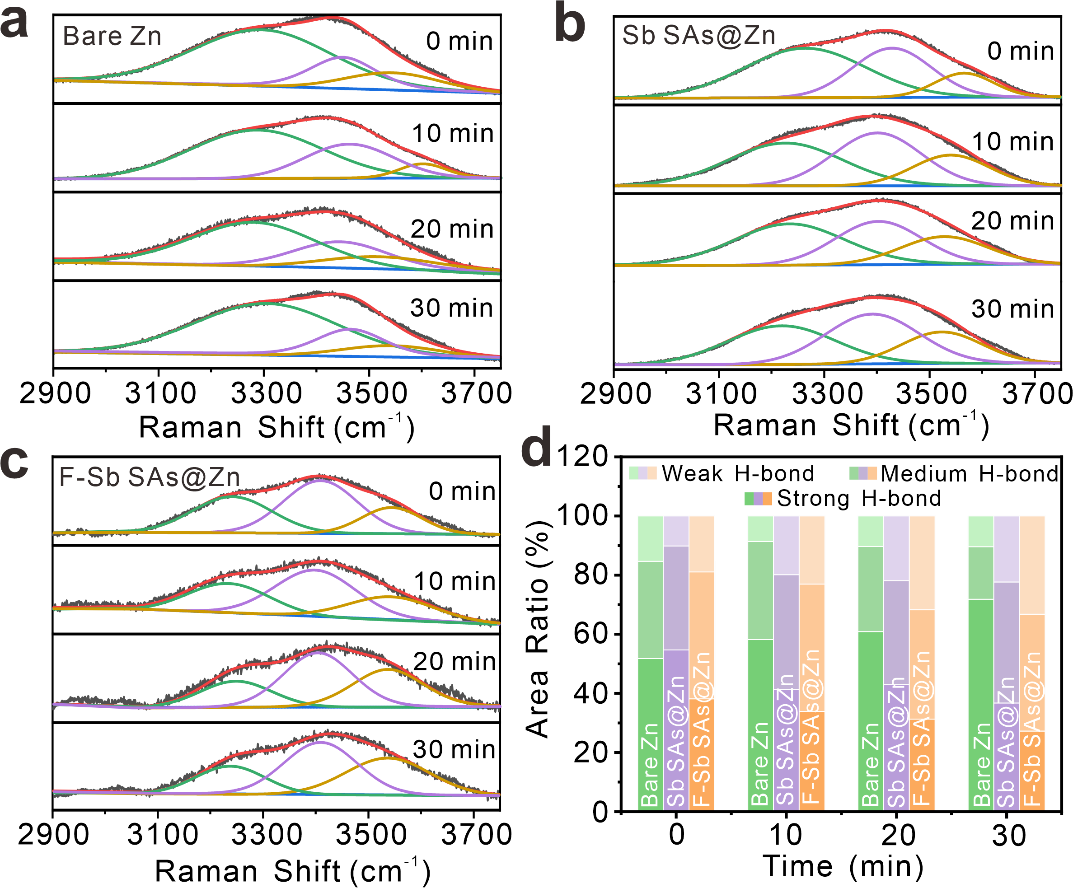
**

**Figure S40**. Fitting Raman spectra of (a) bare Zn, (b) Sb SAs@Zn and (c) F-Sb SAs@Zn between 2900 and 3750 cm^-1^ in the plating process of Zn^2+^. (d) Raman spectral distribution of O-H bonds at various electrode/electrolyte interfaces.

**
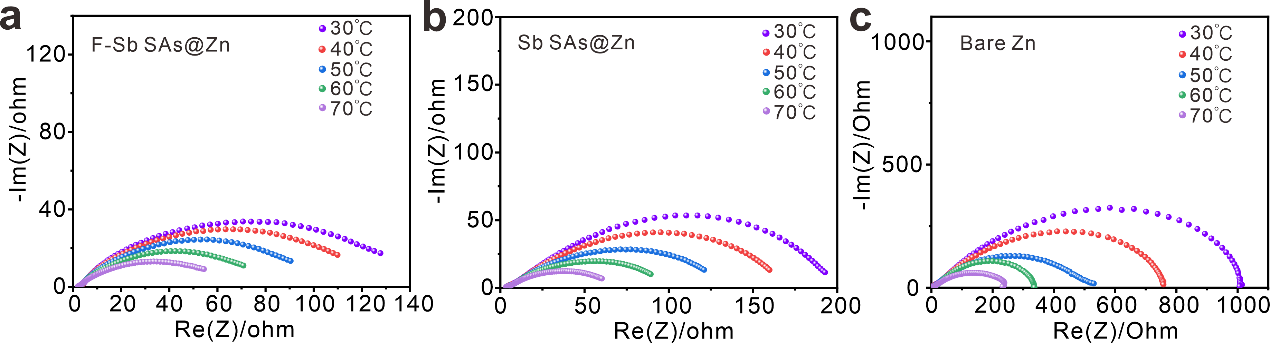
**

**Figure S41**. EIS plots of (a) F-Sb SAs@Zn, (b) Sb SAs@Zn and (c) bare Zn symmetric cells at different temperatures.


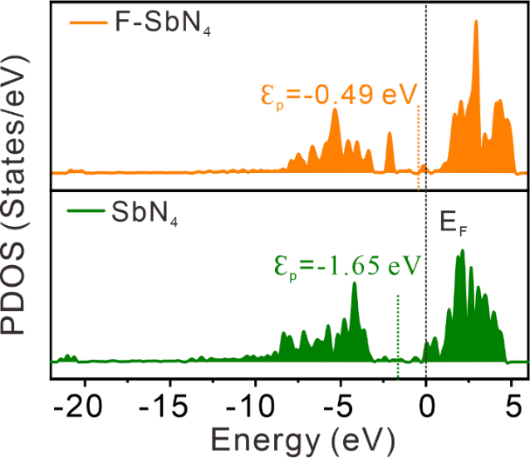


**Figure S42**. The projected density of states (PDOS) of SbN_4_ (bottom) and F-SbN_4_ (upper).


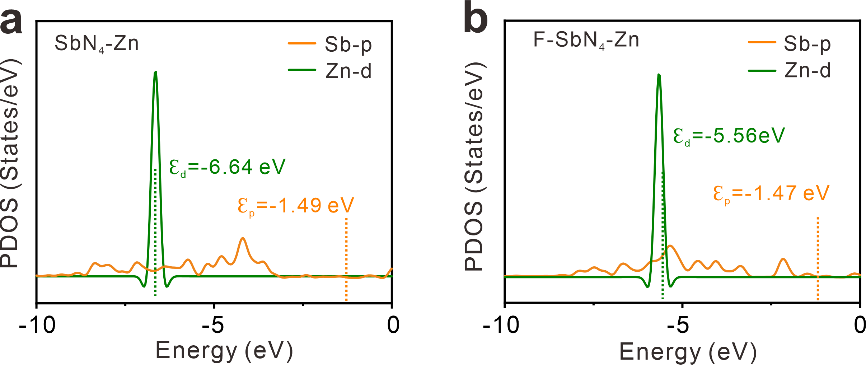


**Figure S43.** The PDOS of (a) SbN_4_ and (b) F-SbN_4_ after Zn adsorption.


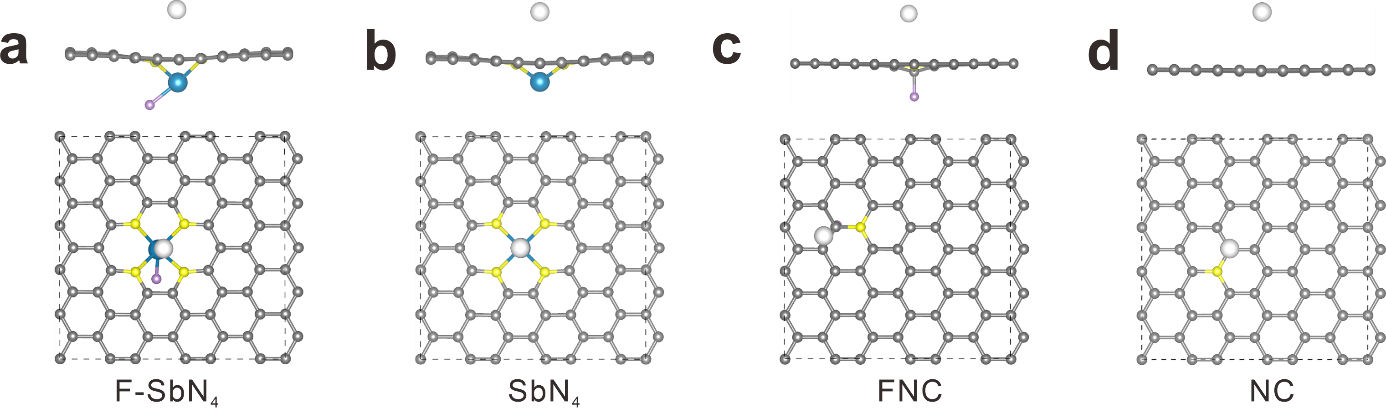


**Figure S44**. The optimized adsorption structure of Zn on the (a) F-SbN_4_, (b) SbN_4_, (c) F,N co-doped carbon and (d) N-doped carbon. The white, grey, blue, yellow, and purple balls represent Zn, C, Sb, N and F atoms, respectively.


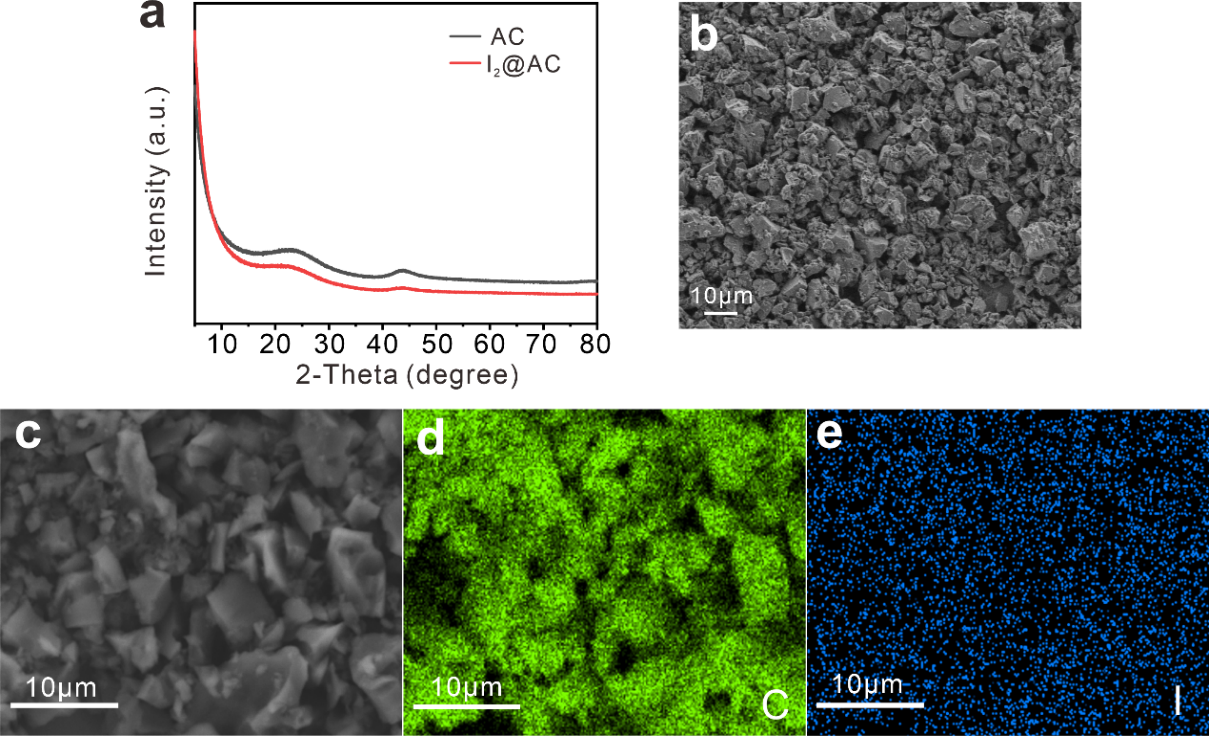


**Figure S45**. (a) XRD patterns of AC and AC@I_2_. SEM images of AC (b), AC@I_2_ (c), and corresponding elemental mapping images of AC@I_2_ (d,e).


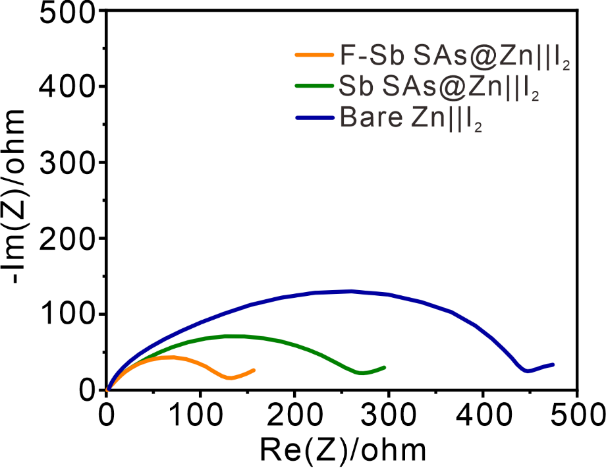


**Figure S46**. EIS plots of Zn-I_2_ batteries before cycling.


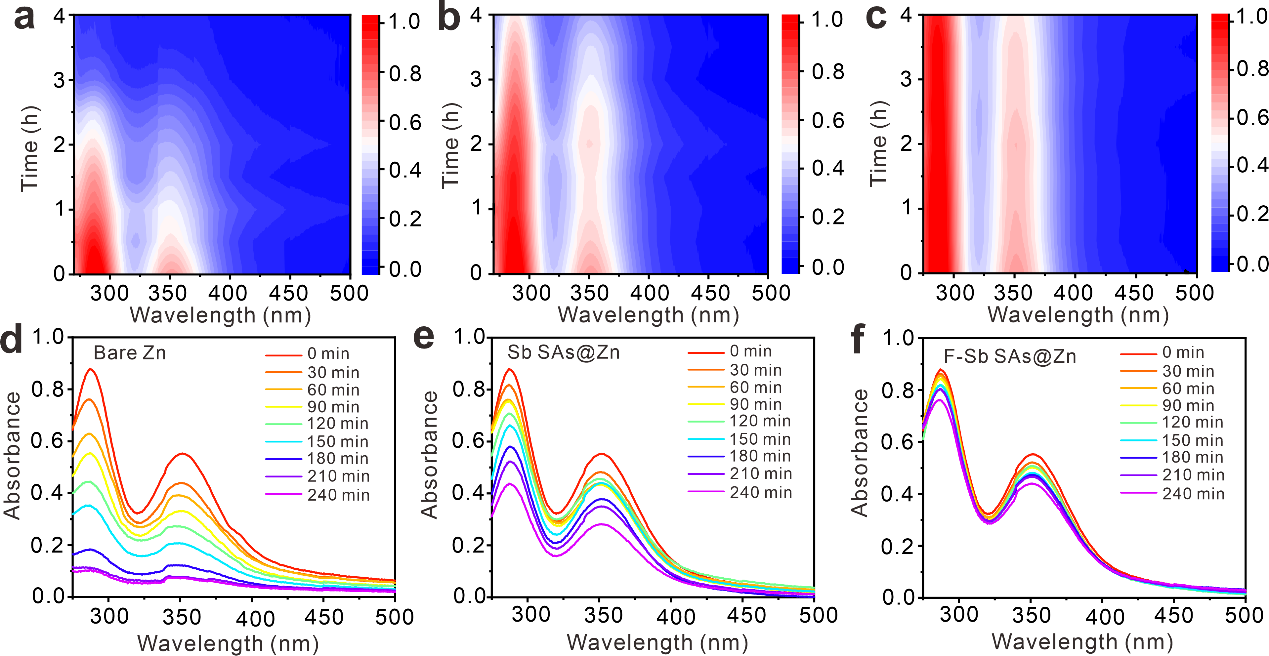


**Figure S47**. In situ UV-vis absorption spectra of I_3_^-^ solution when bare Zn (a, d), Sb SAs@Zn (b, e), and F-Sb SAs@Zn (c, f) are soaked in.


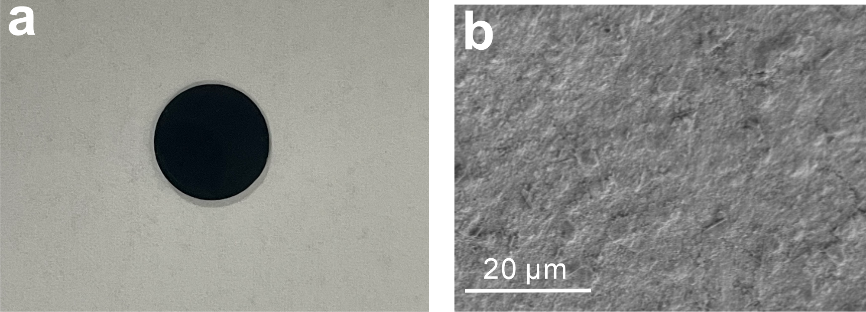


**Figure S48.** (a) Digital picture and (b) SEM image of F-Sb SAs@Zn surface after Zn||I_2_ full-cell cycling.


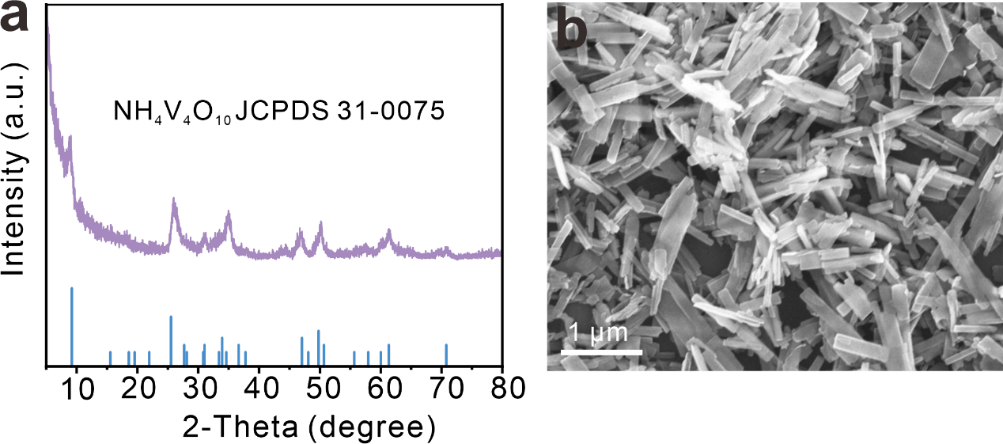


**Figure S49**. (a) XRD pattern and (b) SEM image of NH_4_V_4_O_10_.


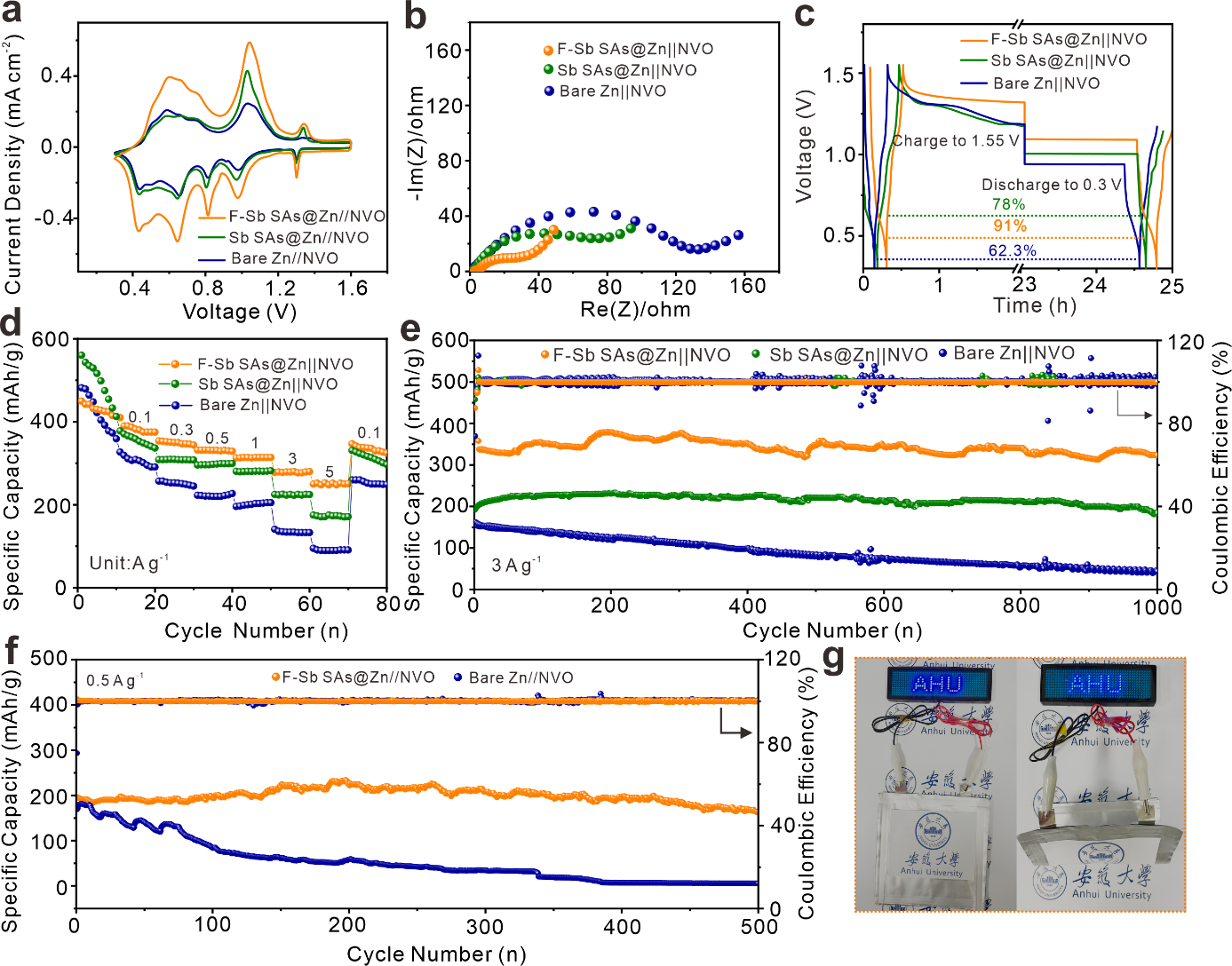


**Figure S50**. (a) CV curves at a scan rate of 0.5 mV s^-1^. (b) EIS plots before cycling. (c) Self-discharging voltage-time curves of bare Zn||NVO, Sb SAs@Zn||NVO and F-Sb SAs@Zn||NVO. (d) Rate performance of bare Zn||NVO, Sb SAs@Zn||NVO and Sb SAs@Zn||NVO full cells. (e) Cycling performance of bare Zn||NVO, Sb SAs@Zn||NVO and F-Sb SAs@Zn||NVO full cells at 3 A g^-1^. (f) Cycling performance of pouch cells based on bare Zn and F-Sb SAs@Zn at 0.5 A g^-1^. (g) Photograph of LED powered by the F-Sb SAs@Zn pouch cell.

**Table S1**. FT-EXAFS fitting parameters at the Sb *L*3-edge. (S_0_^2^=0.88)

| **Samples** | **Scattering**  **pair** | **CN** | ***R* (Å)** | ***σ^2^* (10^-3^Å^2^)** | ***R* factor** |
| --- | --- | --- | --- | --- | --- |
| F-Sb SAs | Sb-N | 4.3 | 2.13 | 2.73 | 0.014 |
| F-Sb SAs | Sb-F | 1.2 | 2.6 | 0 | 0.021 |
| Sb SAs | Sb-N | 3.7 | 2.11 | 6.6 | 0.013 |
| Sb | Sb-Sb | 4.1 | 2.91 | 5.1 | 0.051 |

**CN**, the coordination numbers; ***R***, distance between absorber and backscatter atoms; ***σ^2^***, Debye-Waller factor; ***R*** factor is used to evaluate the degree of fitting. This value was fixed during EXAFS fitting.

**Table S2**. Long-cycle performance comparison of recently reported symmetric cell with modified zinc anode.

| **Anode** | **Electrolyte** | **Current density (mA·cm^−2^)** | **Capacity**  **(mAh·cm^−2^)** | **Cumulative Capacity (mAh·cm^−2^)** | **Cycle life (h)** | **References** |
| --- | --- | --- | --- | --- | --- | --- |
| **F-Sb SAs@Zn** | **2 M ZnSO_4_** | **5** | **1** | **15000** | **6000** | **This work** |
|  |  | **20** | **5** | **12000** | **1200** |  |
| Bi SAs@Zn | 2 M ZnSO_4_ | 5 | 1 | 10500 | 4200 | ^[5]^ |
| ZWO@Zn | 2 M ZnSO_4_ | 1 | 1 | 900 | 1800 | ^[6]^ |
| ZP@Zn | 2 M ZnSO_4_ | 1 | 1 | 1200 | 2400 | ^[7]^ |
| Sn NC@Zn | 2 M ZnSO_4_ | 1 | 1 | 500 | 1000 | ^[8]^ |
| ND@Zn | 2 M ZnSO_4_ | 10 | 5 | 5000 | 1000 | ^[9]^ |
| ZnO/C-Zn | 2 M ZnSO_4_ | 5 | 1 | 1700 | 680 | ^[10]^ |
| Sb@Zn | 2 M ZnSO_4_ | 3 | 1 | 1500 | 1000 | ^[11]^ |
| Ti-Zn | 2 M ZnSO_4_ | 2 | 2 | 2200 | 1100 | ^[12]^ |
| ZnTe@Zn | 2 M ZnSO_4_ | 1 | 0.5 | 1650 | 3300 | ^[13]^ |
| COP-Zn | 2 M ZnSO_4_ | 20 | 1 | 4800 | 480 | ^[14]^ |
| DPM-Zn | 2 M ZnSO_4_ | 1 | 0.5 | 850 | 1700 | ^[15]^ |
| ZrO_2_@Zn | 2 M ZnSO_4_ | 1 | 1 | 3000 | 6000 | ^[16]^ |
| ZSO@Zn | 2 M ZnSO_4_ | 5 | 1 | 3800 | 1520 | ^[17]^ |

**Table S3.** Comparison of energy density in this work with previously reported works.

| **Energy Density (Wh Kg^-1^)** | **Cycle Number (n)** | **References** |
| --- | --- | --- |
| **61.8** | **2000** | **This work** |
| 97.34 | 5000 | ^[18]^ |
| 61 | 1000 | ^[19]^ |
| 54.3 | 1000 | ^[5]^ |
| 51.52 | 100 | ^[20]^ |
| 43.5 | 150 | ^[21]^ |
| 20 | 800 | ^[22]^ |

**References**

[1] G. K. J. Furthmuller, *Efficient iterative schemes for ab initio total-energy calculations using a plane-wave basis set,* *Physical Review B* **1996**, *54(16)*, 11169-11185.

[2] K. B. John P. Perdew, * Matthias Ernzerhof, *Generalized Gradient Approximation Made Simple,* *Physical Review Letters* **1996**, *77(18)*, 3865-3868.

[3] S. Grimme, *Semiempirical GGA‐type density functional constructed with a long‐range dispersion correction,* *Journal of Computational Chemistry* **2006**, *27*, 1787-1799.

[4] a) C. Yang, P. Woottapanit, S. Geng, K. Lolupiman, X. Zhang, Z. Zeng, G. He, J. Qin, *Highly Reversible Zn Anode Design Through Oriented ZnO(002) Facets,* *Advanced Materials* **2024**, *36*, 2408908; b) Y. Meng, M. Wang, J. Xu, K. Xu, K. Zhang, Z. Xie, Z. Zhu, W. Wang, P. Gao, X. Li, W. Chen, *Balancing Interfacial Reactions through Regulating p‐Band Centers by an Indium Tin Oxide Protective Layer for Stable Zn Metal Anodes,* *Angewandte Chemie International Edition* **2023**, *62*, e202308454.

[5] M. Zhang, H. Wei, Y. Zhou, W. Wen, L. Zhang, X.-Y. Yu, *A multi-functional protective material with atomically dispersed zincophilic sites enabling long-life zinc anodes,* *Chemical Science* **2024**, *15*, 18187-18195.

[6] J. Cao, H. Wu, D. Zhang, D. Luo, L. Zhang, X. Yang, J. Qin, G. He, *In‐Situ Ultrafast Construction of Zinc Tungstate Interface Layer for Highly Reversible Zinc Anodes,* *Angewandte Chemie International Edition* **2024**, *63*, e202319661.

[7] Y. Zhang, R. Wang, H. Ao, T. Ma, X. Zhu, X. Zhang, J. Rong, Z. Zhou, Z. Bai, S. X. Dou, N. Wang, Z. Li, *Zn2⁺‐Rich Chelate Layer Facilitates Ultrahigh‐Rate Zinc Anodes Via Cation Compensation and Anion Repulsion,* *Advanced Energy Materials* **2025**, *15*, 2404203.

[8] Y. Wang, Y. Tan, C. Cheng, *Atomic Sn sites on nitrogen-doped carbon as a zincophilic and hydrophobic protection layer for stable Zn anodes,* *Journal of Materials Chemistry A* **2024**, *12*, 428-439.

[9] K. Liu, M. Sun, S. Yang, G. Gan, S. Bu, A. Zhu, D. Lin, T. Zhang, C. Luan, C. Zhi, P. Wang, B. Huang, G. Hong, W. Zhang, *Multifunctional Nanodiamond Interfacial Layer for Ultra‐Stable Zinc‐Metal Anodes,* *Advanced Energy Materials* **2024**, *14*, 2401479.

[10] W. Deng, N. Zhang, X. Wang, *Hybrid interlayer enables dendrite-free and deposition-modulated zinc anodes,* *Chemical Engineering Journal* **2022**, *432*, 134378.

[11] L. Hong, L. Y. Wang, Y. Wang, X. Wu, W. Huang, Y. Zhou, K. X. Wang, J. S. Chen, *Toward Hydrogen‐Free and Dendrite‐Free Aqueous Zinc Batteries: Formation of Zincophilic Protective Layer on Zn Anodes,* *Advanced Science* **2022**, *9*, 2104866.

[12] Y. Zhao, S. Guo, M. Chen, B. Lu, X. Zhang, S. Liang, J. Zhou, *Tailoring grain boundary stability of zinc-titanium alloy for long-lasting aqueous zinc batteries,* *Nature Communications* **2023**, *14*.

[13] R. Wang, S. Xin, D. Chao, Z. Liu, J. Wan, P. Xiong, Q. Luo, K. Hua, J. Hao, C. Zhang, *Fast and Regulated Zinc Deposition in a Semiconductor Substrate toward High‐Performance Aqueous Rechargeable Batteries,* *Advanced Functional Materials* **2022**, *32*, 2207751.

[14] T. Chen, X. Li, Z. Xiang, *Disrupting the Spatiotemporal Coupling of Side Reactions via Zn-Centered Covalent Organic Polymer Enables Highly Reversible Zn Metal Anodes,* *ACS Nano* **2025**, *19*, 24773-24783.

[15] S. Cai, J. Hu, R. Wu, Y. Luo, Y. Xin, G. Zou, H. Hou, X. Ji, *Electro‐Ionic‐Field Regulation through Dipole Molecule Layer toward Dendrite‐Free Zinc Anode,* *Advanced Functional Materials* **2024**, *34*, 2410158.

[16] B. Wei, J. Zheng, Abhishek, X. Liu, J. Wu, Z. Qi, Z. Hou, R. Wang, J. Ma, A. N. Gandi, Z. Wang, H. Liang, *Design Principle of Insulating Surface Protective Layers for Metallic Zn Anodes: A Case Study of ZrO_2_,* *Advanced Energy Materials* **2024**, *14*, 2401018.

[17] R. Guo, X. Liu, F. Xia, Y. Jiang, H. Zhang, M. Huang, C. Niu, J. Wu, Y. Zhao, X. Wang, C. Han, L. Mai, *Large‐Scale Integration of a Zinc Metasilicate Interface Layer Guiding Well‐Regulated Zn Deposition,* *Advanced Materials* **2022**, *34*, 2202188.

[18] H. Wu, J. Hao, S. Zhang, Y. Jiang, Y. Zhu, J. Liu, K. Davey, S.-Z. Qiao, *Aqueous Zinc–Iodine Pouch Cells with Long Cycling Life and Low Self-Discharge,* *Journal of the American Chemical Society* **2024**, *146*, 16601-16608.

[19] W. Ling, C. Nie, X. Wu, X.-X. Zeng, F. Mo, Q. Ma, Z. Lu, G. Luo, Y. Huang, *Ion Sieve Interface Assisted Zinc Anode with High Zinc Utilization and Ultralong Cycle Life for 61 Wh/kg Mild Aqueous Pouch Battery,* *ACS Nano* **2024**, *18*, 5003-5016.

[20] S. Liu, J. Vongsvivut, Y. Wang, R. Zhang, F. Yang, S. Zhang, K. Davey, J. Mao, Z. Guo, *Monolithic Phosphate Interphase for Highly Reversible and Stable Zn Metal Anode,* *Angewandte Chemie International Edition* **2022**, *62*, e202215600.

[21] Q. Zhang, Y. Ma, Y. Lu, X. Zhou, L. Lin, L. Li, Z. Yan, Q. Zhao, K. Zhang, J. Chen, *Designing Anion‐Type Water‐Free Zn^2+^ Solvation Structure for Robust Zn Metal Anode,* *Angewandte Chemie International Edition* **2021**, *60*, 23357-23364.

[22] S. Zhang, J. Chen, W. Chen, Y. Su, Q. Gou, R. Yuan, Z. Wang, K. Wang, W. Zhang, X. Hu, Z. Zhang, P. Wang, F. Wan, J. Liu, B. Li, Y. Wang, G. Zheng, M. Li, J. Sun, *Regulating Water Molecules via Bioinspired Covalent Organic Framework Membranes for Zn Metal Anodes,* *Angewandte Chemie International Edition* **2025**, *64*, e202424184
